# Fibroblast Biomarkers of Sporadic Parkinson’s Disease and LRRK2 Kinase Inhibition

**DOI:** 10.1007/s12035-015-9435-4

**Published:** 2015-09-23

**Authors:** G. A. Smith, J. Jansson, E. M. Rocha, T. Osborn, P. J. Hallett, O. Isacson

**Affiliations:** Neuroregeneration Research Institute, McLean Hospital/ Harvard Medical School, 115 Mill Street, Belmont, 02478 USA

**Keywords:** Sporadic Parkinson’s disease, Mitochondria, Biomarkers, LRRK2, Fibroblasts, Parkin

## Abstract

It has been uncertain whether specific disease-relevant biomarker phenotypes can be found using sporadic Parkinson’s disease (PD) patient-derived samples, as it has been proposed that there may be a plethora of underlying causes and pathological mechanisms. Fibroblasts derived from familial PD patients harboring leucine-rich repeat kinase 2 (LRRK2), PTEN-induced putative kinase 1 (PINK1), and Parkin mutations show clear disease-relevant mitochondrial phenotypes, which are exacerbated under conditions of pharmacological stress. We utilized fibroblasts derived from non-familial sporadic PD patients (without LRRK2 mutations) or LRRK2 mutation carriers to directly compare the cellular phenotypes during and after mitochondrial stress. We then determined the effects of pharmacological LRRK2 kinase inhibition using LRRK2-in-1. We found that there were two distinct populations of sporadic PD patient-derived fibroblast lines. One group of sporadic PD lines was highly susceptible to valinomycin-induced mitochondrial depolarization, emulating the mutant LRRK2 phenotype. These lines showed elevated mitochondrial superoxide/ nitric oxide levels, displayed increased mitochondrial and lysosome co-localization, and an increased rate of mitochondrial collapse, which corresponded with changes in mitochondrial fission and fusion proteins. The application of LRRK2-in-1 reversed decreased levels of mitochondrial and lysosome co-localization and partially restored mitochondrial network associated proteins and the mitochondrial membrane potential in the fibroblasts. This study identifies novel mitochondrial biomarkers in sporadic PD patient-derived fibroblast lines, which could be used as preclinical tools in which to test novel and known neuroprotective compounds.

**Electronic supplementary material:**

The online version of this article (doi:10.1007/s12035-015-9435-4) contains supplementary material, which is available to authorized users.

## Introduction

Sporadic Parkinson’s disease (PD) accounts for 90 % of the global PD population [1] and there is likely to be a large number of underlying causes [2–5]. This may pose a significant problem in testing new treatments at clinical trial as a “one size fits all” approach may yield few significant findings. Therefore, it is imperative to test therapeutics in easily accessible tissues, such as fibroblasts, from sporadic PD patients to determine if and how they will respond to novel treatments. For example, there has been much debate over whether leucine-rich repeat kinase 2 (LRRK2) kinase inhibition, using small molecules, would be beneficial in sporadic cases [6] and consequently, only LRRK2 mutation carriers have been recruited to clinical trial. To test such therapeutics, a reliable and robust readout of cellular function must be achieved for the analysis of sporadic PD cases on an individual basis. We therefore hypothesized that specific phenotypes observed using fibroblasts, obtained by a skin biopsy, could be used as a diagnostic tool for the ability of novel treatments to prevent mitochondrial phenotypes observed in the prodromal phase. There has been much focus on investigating prodromal changes in mouse models of PD [7] and finding new biomarkers in human cell lines will inevitably broaden the tools available for testing novel therapeutics.

Familial and sporadic PD-derived fibroblast lines show disease-relevant changes in mitochondrial-associated gene expression profiles [8]. In addition, we and others have characterized mitochondrial phenotypes in fibroblast cell lines harboring LRRK2 [9–14], PTEN-induced putative kinase 1 (PINK1) [8, 10, 15], and Parkin [16–20] mutations, both at baseline and under conditions of pharmacological stress. Specifically, mutant LRRK2 carrying fibroblast lines have been instrumental to investigate mitochondrial phenotypes and hence, have recently been used for exploratory drug screens [21]. The expression of the G2019S LRRK2 mutation in fibroblast cells was found to be associated with mitochondrial uncoupling [13], excessive fission [14], elevated autophagy levels [14], and mitochondrial depolarization [13]. Despite these clear mitochondrial phenotypes, little is known about how LRRK2 functions in this context [22]. Recently, it has been determined that LRRK2 and the mitochondrial fission protein dynamin-like protein 1 (DLP1) can interact in neurons [23, 24]. Levels of DLP1 are increased in SH-SY5Y-derived cells following the expression of mutant LRRK2 (G2019S and R1441C) [24]. Furthermore, dominant negative DLP1 can mitigate this phenotype [14]. It is currently unknown whether this mechanism relies on the phosphorylation state of LRRK2 and whether currently available LRRK2 kinase inhibitors could rescue this fission phenotype. The role of LRRK2 in sporadic PD is poorly understood. Recently, a proteomic screen has revealed critical LRRK2 interacting proteins, known to affect Golgi clearance by autophagy [25]. Two of these proteins, GAK and Rab7L1, have been identified in risk loci by GWAS-based screening of sporadic PD patients [25]. This suggests that LRRK2-mediated pathway dysregulation may link sporadic and familial PD. Sporadic PD patient-derived fibroblast lines were recently shown to be more vulnerably to rotenone toxicity [26]. Under this paradigm, cells displayed deficits in the ubiquitin proteasome system and mitochondrial respiration; however, autophagy and redox changes could not be detected [26]. The specific biological processes controlled by LRRK2 have yet to be investigated in sporadic PD-derived fibroblast cell lines.

We have previously investigated induced pluripotent stem cells (iPSCs) derived from dermal fibroblasts from PD patients harboring mutations in LRRK2 and PINK1 and differentiated to neural cells, following treatment with specific small molecules such as valinomycin, oligomycin, CCCP, and rotenone that are specifically detrimental to mitochondrial function [10]. The depolarization of mitochondria caused by the K+ ionophore and valinomycin, caused similar responses in familial PD patient-derived fibroblast lines and neural cells [10], and hence, is likely the most relevant agent in which to exacerbate phenotypes in sporadic fibroblast lines. Preventing mitochondrial depolarization as the result of impaired clearance, complex 1 inhibition, defective fission/fusion events, calcium homeostasis deficits, and oxidative stress [27] is a reasonable therapeutic pathway for PD.

In these experiments, we utilized fibroblasts derived from sporadic PD patients and LRRK2 mutation carriers and directly compared the phenotypes observed following mitochondrial stress and the beneficial effect of pharmacological LRRK2 kinase inhibition. The LRRK2-in-1 compound was chosen to test in this system, as it is one of the few compounds that targets both the mutant and wild-type forms of LRRK2 [28] and hence, would be applicable for the treatment of both LRRK2 mutation-carrying and sporadic PD patients. This study proposes a number of cell stress biomarker phenotypes, which could be used as preclinical diagnostic tools in which to test novel and known compounds for their ability to ameliorate mitochondrial deficits.

## Materials and Methods

### Fibroblast Lines and Sequencing

Sporadic PD patient (*N* = 10) and healthy subject control (*N* = 14)-derived fibroblast cell lines were obtained from Coriell and the NIA Aging Cell biorepositories (ID numbers are indicated in Supp. Table 1- online resource). Sporadic PD patient and healthy subject control-derived fibroblast cell lines were age and gender matched where possible. Mutant LRRK2 (G2019S *N* = 4 and R1441C *N* = 4)-derived fibroblast lines were used were either previously described [10] or obtained from the Mayo Clinic. Sporadic PD patient-derived fibroblast lines were obtained from patients that did not have a family history of PD and sequencing results indicated that they did not have mutations in LRRK2 or GBA1 genes. Sequencing was carried out at the Harvard Medical School Translational Genomics Core (Cambridge, MA) using Nextera PCR (for LRRK2) and MiSeq (for GBA1) systems *(Illumina)*.

### Cell Culture

PD patient and healthy subject control-derived fibroblast lines were cultured in standard medium containing DMEM (Gibco), 10 % FBS (Hyclone), and 1 % Penicillin-streptomycin (Gibco #10378-016), 0.5 % glutamine (Gibco), and 1 % non-essential amino acids (Gibco). Cell cultures were incubated at 37 °C and 5 % CO_2_. Cells were grown on 10-cm dishes and passaged twice per week or when confluence reached approximately 90 %. For each passage, cells were washed in PBS and dissociated using 0.05 % Trypsin (Gibco). Cell passage numbers did not exceed 20.

### Pharmacological Compounds

For pharmacological assays, cells were incubated with valinomycin (Sigma) in standard cell culture media with 0.1 % DMSO at range of 0.1 to 200 μM. 0.1 % DMSO was added to the culture media for control conditions. The LRRK2 kinase inhibitor LRRK2-in-1 was co-administered with valinomycin (10 μM) at ranges of 1–30 μM in 0.1 % DMSO.

### LDH Release Assay

In vitro toxicity was determined by lactate dehydrogenase (LDH) assay (Roche) to measure the conversion of a tetrazolium substrate by LDH enzyme released through the cell plasma membrane during cell death. Fibroblasts were placed in a clear, flat-bottomed 96-well plate at a density of 5000 cells per well. Two days post plating, cells were incubated at 37 **°**C with either valinomycin, a combination of valinomycin, and LRRK2-in-1 or vehicle. The dose of LRRK2-in-1 was tested to avoid drug-mediated toxicity. The substrate and catalyst were applied according to the manufacturer’s instructions. The LDH-based colorimetric change was analyzed according to the manufacturer’s instructions using the Spectra Max Plus 384 spectrophotometer and Soft Max Pro 5.4.4 software. The optical density of each line was subtracted from a lyzed control for that same line and samples were run in triplicate.

### Nitric Oxide and Superoxide Analysis

Fibroblasts were plated at a density of 5000 cells per well in 96-well plates containing for 48 h. Cells were treated small molecules or vehicle and incubated for 24 h. DAF-FM diacetate (4-amino-5-methylamino-2′,7′-difluorofluorescein diacetate) (Life technologies) and MitoSOX™ Red (Life technologies) were used for the detection of nitric oxide and superoxide, respectively. Then, 10 μM of DAF-FM diacetate was applied to the media containing small molecules for 1 h at 37 °C. Cells were washed in media and left for 30 min at 37 °C to allow for complete de-esterification of the diacetates and imaged immediately. MitoSOX™ Red was applied to cells at a 5-μM working solution in the media containing the small molecules. Fibroblasts were incubated at 37 °C for 10 min, washed in media, and imaged immediately. Six images were taken per well using the IncuCyte ZOOM live cell imager (Essen Bioscience) at 37 °C. The average fluorescent intensity of DAF-FM and MitoSOX™ Red was determined for each image using IncuCyte ZOOM software (Essen Bioscience) and averaged for each well. Samples were run in triplicate.

### Mitochondrial Membrane Potential and Toxicity Assays

Fibroblasts were plated at a density of 20,000 cells per well in 24-well plates containing glass coverslips for 48 h. Cells were treated with small molecules or vehicle and incubated for 24 h, where the confluency was approximately 80 %. The HCS Mitochondrial Health Kit (Life Technologies) was applied to fibroblast-seeded coverslips to simultaneously measure mitochondrial membrane potential and cytotoxicity, using the MitoHealth stain and Image-iT® DEAD™ Green, respectively. MitoHealth stain and Image-iT® DEAD™ Green dyes were prepared in DMSO according to the manufacturer’s instructions and were added to the media for 30 min. The media was removed and cells were fixed using 4 % paraformaldehyde (PFA) for 15 min. Cells were then washed 3× in PBS and incubated in Hoechst (1 mg/200 mls) for 4–5 min. Cells were washed a further 3× in PBS and coverslips were mounted onto slides using an aqueous mountant. Confocal images were taken using the Zeiss LSM 510 meta confocal microscope and Zen software (2009).

### Immunocytochemistry

Cells were fixed by removing medium and adding 4 % PFA for 25 min, followed by three washes in PBS. For immunocytochemistry, PFA-fixed fibroblast-seeded coverslips were washed 3× in PBS and placed in 10 % normal goat serum with 0.3 % Triton X-100 in PBS for 1 h. Coverslips were incubated overnight at room temperature in blocking solution with anti-Tom20 (Santa Cruz 1:100) and anti-LAMP1 (Abcam 1:100) or anti-Parkin (Abcam 1:100). Coverslips were washed 3× in PBS and incubated with secondary antibodies in PBS for 1 h: Alexa Fluor 488 (Life Technologies, 1:500) and Alexa Fluor 568 (Life Technologies, 1:500). Coverslips were washed 3× in PBS and incubated in Hoechst (1 mg/200 mls) for 4–5 min and washed a further 3× in PBS. Coverslips were then mounted onto slides using an aqueous mountant. Confocal images were taken using the Zeiss LSM 510 meta confocal microscope and Zen software (2009).

### Live Cell Imaging of Mitochondria

Fibroblasts were plated at a density of 5000 cells per well in 96-well plates containing for 48 h. Cells were treated with small molecules or vehicle and incubated for 24 h. MitoTracker® Red FM (Life Technologies) was used to visualize mitochondria over 36 h. MitoTracker® Red FM was reconstituted in DMSO to a concentration of 1 mM and diluted in media to a final working concentration of 300 uM. Cells were incubated at 37 °C for 30 mins and the media replaced. The new media added contained either vehicle (0.1 % DMSO) or valinomycin (10 μM). Fibroblasts lines were imaged immediately. Three images were taken per well, every hour, using the IncuCyte ZOOM live cell imager (Essen Bioscience) at 37 °C and 20× magnification. The average phase contrast confluency and red object confluency was determined for each image using IncuCyte ZOOM software (Essen Bioscience) and averaged for each well. Phase contrast and red object confluency was determined using user-defined masks (Supp. Fig. 3 - online resource). The red object confluency was normalized to the phase contrast confluency and the data represented as relative change in normalized red object confluency compared to 0 h.

### Immunoblotting

Cells were washed in PBS, dissociated with 0.05 % trypsin, pelleted and resuspended in standard cell culture media. Cells were pelleted, excess media removed and snap frozen on dry ice. Cell lysates were resuspended in ice-cold buffer containing: 300 mM sucrose in TE buffer (Bio-Rad), Phosphatase inhibitors I and II (1:100), proteinase inhibitors (1:100) (Thermo Halt proteinase inhibitor single use cocktail), EDTA, and 1 % Triton X-100. Cell lysates were sonicated with three short pulses. A sample of the supernatant was reserved for protein content determination (BCA Assay, Pierce) and the remaining solution was stored at −80 °C until further use. Then, 80 mg of each prepared protein sample were loaded into the criterion precast 4–12.5 % SDS polyacrylamide gels (Bio-Rad) and protein samples were run using the Bio-Rad system. Proteins were then transferred from the gel to a PVDF membrane by an electrical charge of 21 V and 2.5 amps for 7 mins. Membranes were washed 5× in Tris-buffered saline with 0.1 % Tween 20 (TBS-T) and blocked using in 5 % protein blocker (Bio-Rad) in TBS-T. Membranes were then incubated overnight at 4 °C with the following primary antibodies: anti-LRRK2 (Abcam 1:2000, C41-2, ab133474), anti-pLRRK2 S955 (Abcam 1:1000), anti-pLRRK2 S935 (Abcam 1:1000), anti-pLRRK2 S973 (Abcam 1:500), anti-Parkin (Abcam 1:1000), anti-Parkin Cell Signaling (1:1000), anti-PINK1 (Abcam 1:1000), anti-OPA1 (Abcam 1:500), anti-MARCH5 (Abcam 1:1000), anti-Drp1 (Abcam 1:1000), anti-Mfn1 (Origene 1:500), anti-Mfn2 (Abcam 1:500) and anti-GAPDH (Millipore, 1:5000). Blots were also probed using the total OXPHOS human WB Antibody Cocktail (Abcam 1:500). After washing the blots 3× in TBS-T, HRP-conjugated secondary antibodies were then applied for 1 h at room temperature in 5 % protein blocker (Bio-Rad) in TBS-T. The blots were then washed 3× TBS-T, submersed into an ECL-Plus solution (Amersham Biosciences) and exposed using ChemiDoc^TM^ XRS with Image Lab^TM^ software. Optical density analysis was performed with ImageJ software (Version 1.46r) and was used to determine the relative abundance of each protein of interest. Bands were normalized to GAPDH for that same sample and gel. When analyzing phosphorylated LRRK2 levels, bands were normalized to levels of non-phosphorylated LRRK2.

### Quantification and Statistics

One-way ANOVA and multivariate ANOVAs with Bonferroni. Dunnett’s post hoc tests were used to determine differences between fibroblast genotypes and drug treatment conditions. All analyses were conducted using GraphPad Prism (version 5.0) (GraphPad Software, Inc). Statistical significance was determined at the alpha level of 0.05.

Form factor analysis was used to determine the degree of mitochondrial network connectivity and branching in fibroblast lines, as done previously [18, 20]. Photomicrographs of Tom20 stained fibroblasts were taken, converted to gray scale and binarized using ImageJ software (Version 1.46r). A contour was drawn around individual fibroblasts (*N* = 25/group) and the binarized image was run through the form factor analysis plug-in. Form factor was defined as (Pm2)/(4πAm), where Pm is the length of mitochondrial outline and Am is the area of mitochondria.

Co-localization analysis was used to determine the percentage of mitochondria in lysosomes per cell, as a read out of mitophagy. Raw images of Tom20 and LAMP1 stained fibroblasts were taken and were run through the co-localization plug-in using ImageJ software (version 1.46r). The plug-in required photomicrographs in two color channels and generated an 8-bit image with only the colocalized points between the two channels. The plug then combined the three 8-bit images (red, green, and co-localization in white) to a single RGB image. Representative photomicrographs are indicated in Supp. Fig. 8 - online resource. The percent area of co-localization was measured on threshold images using the analyze particles function and expressed as a percentage of the total area of Tom20 staining.

## Results

### A Group of Sporadic PD Patient Derived Fibroblast Are Vulnerable to Mitochondrial Stress

Mitochondrial dysfunction is widely recognized as a trigger of both sporadic and familial forms of PD and mitochondrial abnormalities are observed in post mortem tissue from PD patients [6, 29, 30]. Mitochondrial dysfunction likely plays a role in the manifestation of the disease later in life. Recapitulating the impact of “mitochondrial aging” in vitro*,* using juvenile cells has been challenging; however, we and others have previously shown robust phenotypes of LRRK2, PINK1, and Parkin mutant PD patient fibroblasts when subjected to low doses of small molecules which induce specific mitochondrial stress [10, 11, 18, 20]. In order to determine the unique features of individuals with sporadic PD, we use fibroblast samples (Supp. Table 1- online resource) as a methodological tool to determine a number of mitochondrial stress-related biomarkers, which can be analyzed following the application of therapeutics. Sporadic PD patient-derived fibroblast lines were age and gender matched to healthy subject controls and following sequencing analysis were confirmed to be both LRRK2 and GBA1 mutation negative. Mitochondrial stress, induced by escalating doses of valinomycin caused divergent responses to the fibroblast vulnerability profiles observed between patients harboring LRRK2 mutations (G2019S and R1441C) and sporadic PD lines, when compared to healthy subject control lines, as analyzed by a lactate dehydrogenase (LDH) colorimetric assay (Supp. Fig. 1- online resource). There was a significant interaction between dose and patient source (group × dose, *F*_20.140_ = 13.97, *p* < 0.001), which was clearly attributable to the sensitive subgroup of idiopathic PD patients, who showed significantly heightened sensitivity at all mid-range doses, but merging with the controls and less-sensitive controls at the highest doses. However, for a subgroup of the sporadic PD patients there was no statistical interaction, resembling healthy subject controls. We therefore grouped patient fibroblasts that were “highly sensitive” or “normally sensitive” for all other analyses to determine fundamental mitochondrial-related differences between these groups. There was no statistical difference in the mean age-of-onset (*T*_2,8_ = 0.08, *p* = n.s.) and age-of-biospy (*T*_2,8_ = 0.03, *p* = n.s.) between highly sensitive and normally sensitive sporadic PD groups. Groups were also matched for gender (highly sensitive = 2 F and 3 M; normally sensitive = 2 F and 3 M). Specifically, highly sensitive sporadic PD lines showed a heightened toxicity to valinomycin treatment at doses of 20 μM (*p* < 0.001), 40 μM (*p* < 0.01), and 50 μM (*p* < 0.05) compared to healthy subject controls, which was equivalent to the mutant LRRK2 (G2019S) cell death profile (Supp. Fig. 1- online resource).

Using live cell imaging, we also determined the rate of mitochondrial collapse around the perinuclear cellular compartment, induced by 10 μM of valinomycin over 36 h (Supp. Fig. 2a - online resource), using user-defined masks for confluency and red object area (Supp. Fig. 3 - online resource). Mitochondrial collapse occurred at a faster rate in more sensitive sporadic PD and LRRK2 mutation (R1441C) lines (Supp. Fig. 2b - online resource ), most evident at 12 h (*p* < 0.001), (Supp. Fig. 2c - online resource). An increase in nitric oxide species was observed under vehicle conditions in mutant LRRK2 (G2019S) (*p* < 0.05) and highly sensitive sporadic PD lines (*p* < 0.01), yet levels were not exacerbated further by valinomycin treatment (Supp. Fig. 4a, b - online resource). In contrast, superoxide levels did not differ between groups under vehicle conditions and were increased in all PD lines following valinomycin exposure (*p* < 0.05), (Supp. Fig. 4c ,d - online resource). The increase in nitric oxide species in sporadic PD patient-derived fibroblast lines at 10 μM of valinomycin was positively correlated with valinomycin-induced toxicity observed at 50 μM (*p* < 0.05), (Supp. Fig. 4e - online resource). No correlation was observed between valinomycin-mediated cell death and superoxide levels (Supp. Fig. 4f - online resource). However, superoxide levels and nitric oxide levels in sporadic PD patient-derived fibroblast lines were positively correlated (*p* < 0.05) (Supp. Fig. 4g - online resource), indicating a sporadic PD patient fibroblast line specific increase in reactive oxygen species.

### The Effect of LRRK2-in-1 on Valinomycin-Treated Sporadic PD Lines

**Fig. 1:**
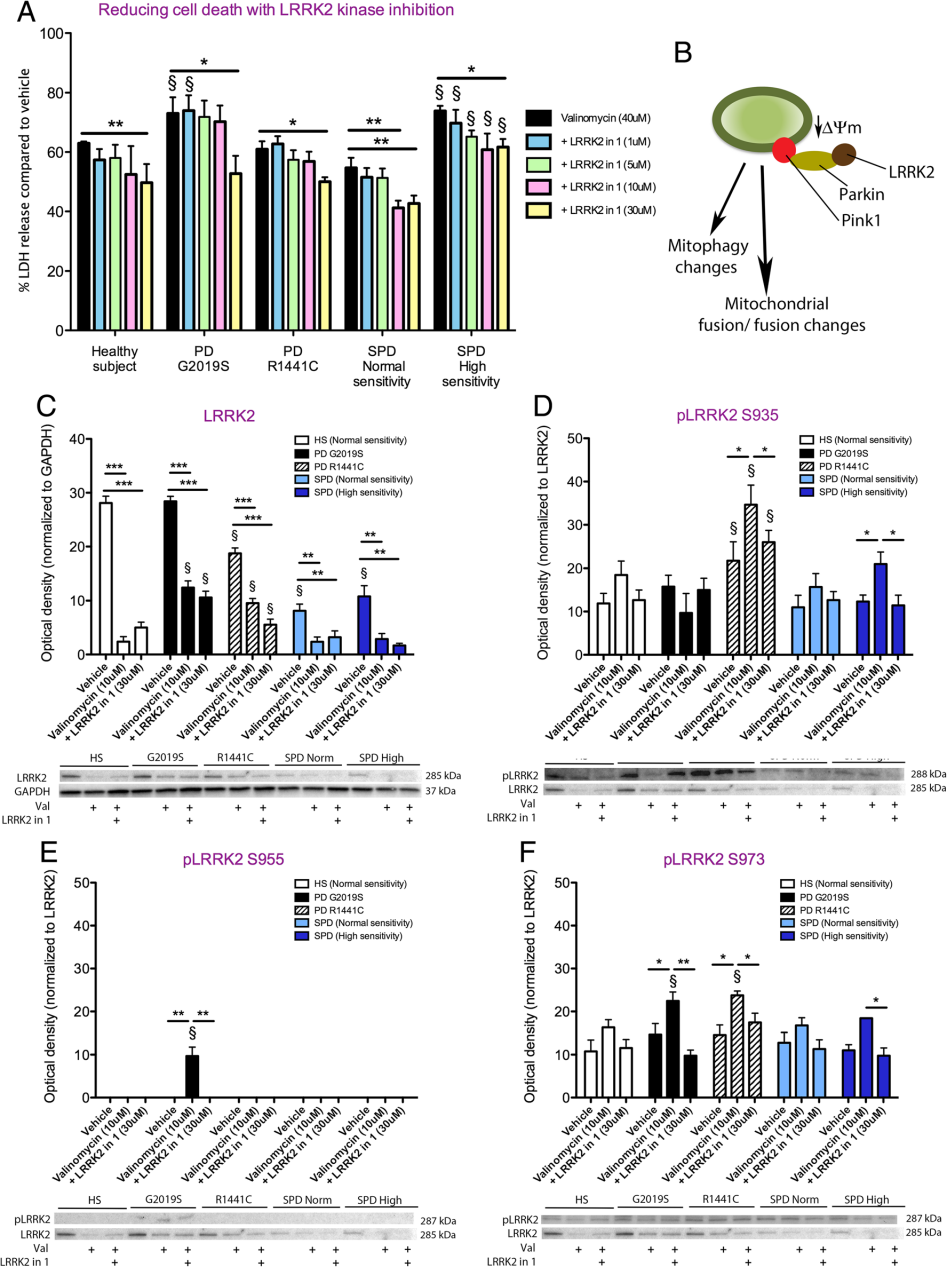
PD patient fibroblast line vulnerability profile to the mitochondrial stressor valinomycin can be rescued by LRRK2-in-1 and is associated with reduced LRRK2 and phosphorylated LRRK2 protein levels. Following the application of valinomycin (40 μM) for 24-h mutant LRRK2 (G2019S) and highly sensitive sporadic PD patient fibroblast lines showed and increase in toxicity, as observed by an LDH release assay, compared to healthy subject control lines (**a**). The co-application of valinomycin (40 μM) and LRRK2-in-1 (10–30 μM) for 24 h reduced toxicity in all cells lines and the preferential increase in toxicity observed in mutant LRRK2 (G2019S) and highly sensitive sporadic PD patient fibroblast lines (**a**). A schematic diagram showing how levels of LRRK2 may modulate the PINK1/ Parkin initiated mitophagy pathway and fission/ fusion events under conditions of valinomycin exposure (**b**). LRRK2 protein levels were reduced in mutant LRRK2 (R1441C) and sporadic PD patient fibroblast lines at baseline and were decreased further in all lines following valinomycin (10 μM) application (**c**). LRRK2-in-1 (30 μM) did not alter LRRK2 protein levels compared to valinomycin only conditions in any of the fibroblast lines (**c**). The level of pLRRK2 (S935) was increased in the mutant LRRK2 (R1441C) line under vehicle conditions (**d**). Valinomycin exposure for 24 h caused an increase in pLRRK2 (S935) in healthy subject control, mutant LRRK2 (R1441C) and sensitive sporadic PD fibroblast lines (**d**). Increased levels of pLRRK2 (S935) could be reversed by LRRK2-in-1 treatment (**d**). pLRRK2 (S955) was only observed in mutant LRRK2 (G2019S) fibroblast lines and protein levels were increased following valinomycin exposure and decreased by the application of LRRK2-in-1 (**e**). Protein levels of pLRRK2 (S973) were also increased by valinomycin treatment in mutant LRRK2 (R1441C and G2019S) and sensitive sporadic PD fibroblast lines (**f**). Heightened levels of pLRRK2 (S973) were reversed to baseline levels following LRRK2-in-1 treatment (**f**). Graphs are annotated as ^§^ *p* < 0.01 compared to healthy subject controls and *p < 0.05, **p < 0.01, and ***p < 0.001 compared to vehicle conditions, as analyzed by a multivariate ANOVAs with Dunnett’s post hoc tests. Graphs are expressed at mean ± SEM. *N* = 4–14/group for LDH assay and *N* = 3/group for Western blot analysis

LRRK2-in-1 has been shown to efficiently reduce LRRK2 kinase activity [28, 31–34], and is being tested in an ongoing phase 1 clinical trial for LRRK2 mutation-carrying PD patients (clinical trial number NCT01424475; no affiliation to the authors). We show that 30 μM of LRRK2-in-1 efficiently reduces the vulnerability of fibroblasts lines to the mitochondria stressor valinomycin (*p* < 0.05) (Fig. 1a). LRRK2-in-1 specifically reduces the exacerbated vulnerability profile of mutant LRRK2 (*p* < 0.05) and highly sensitive sporadic PD patient lines (*p* < 0.05) to valinomycin (Fig. 1a). Given these changes, we decided to quantify the effects of LRRK2-in-1 on LRRK2 and phosphorylated (p)LRRK2, under conditions of mitochondrial stress, which may effect mitophagy, autophagy, and fusion/fission in the sporadic PD fibroblasts (Fig. 1b). Mutant LRRK2 (R1441C) fibroblast lines and sporadic PD patient lines displayed less non-phosphorylated LRRK2 protein levels at baseline conditions (*p* < 0.05), in comparison to healthy subject controls (Fig. 1c). Valinomycin exposure caused a non-cell line-dependent decrease in non-phosphorylated LRRK2 protein levels, which was not rescued by LRRK2-in-1 (Fig. 1c). We next validated the action of LRRK2-in-1 on LRRK2 kinase activity by analyzing levels of pLRRK2 species normalized to non-phosphorylated LRRK2 protein levels. Valinomycin caused a significant increase in the protein levels of pS935 in mutant LRRK2 G2019S (*p* < 0.05) and sensitive sporadic PD fibroblast lines (*p* < 0.05), which were significantly reduced by LRRK2-in-1 treatment (*p* < 0.05), (Fig. 1d). Sporadic PD lines were not associated with LRRK2 phosphorylation at S955 following valinomycin exposure and an increase was only observed in mutant LRRK2 G2019S fibroblast lines, (*p* < 0.05), (Fig. 1e). Nevertheless, the administration of LRRK2-in-1 caused a significant reduction of pS955 LRRK2 species in these lines (*p* < 0.05), (Fig. 1e). Valinomycin exposure also caused an increase in pS973 LRRK2 levels in mutant LRRK2 G2019S (*p* < 0.05), mutant LRRK2 R1441C (*p* < 0.05) and sensitive sporadic PD fibroblast lines (*p* < 0.05), (Fig. 1f). Levels of LRRK2 phosphorylation at S973 were also decreased to baseline levels following LRRK2 kinase inhibition in mutant LRRK2 G2019S (*p* < 0.01), mutant LRRK2 R1441C (*p* < 0.05) and sensitive sporadic PD fibroblast lines (*p* < 0.05), (Fig. 1f).

### LRRK2-in-1 Treatment Modulates Mitochondria Dynamics and Mitochondrial Activity in Sporadic PD Patient-Derived Fibroblast Lines

**Fig. 2:**
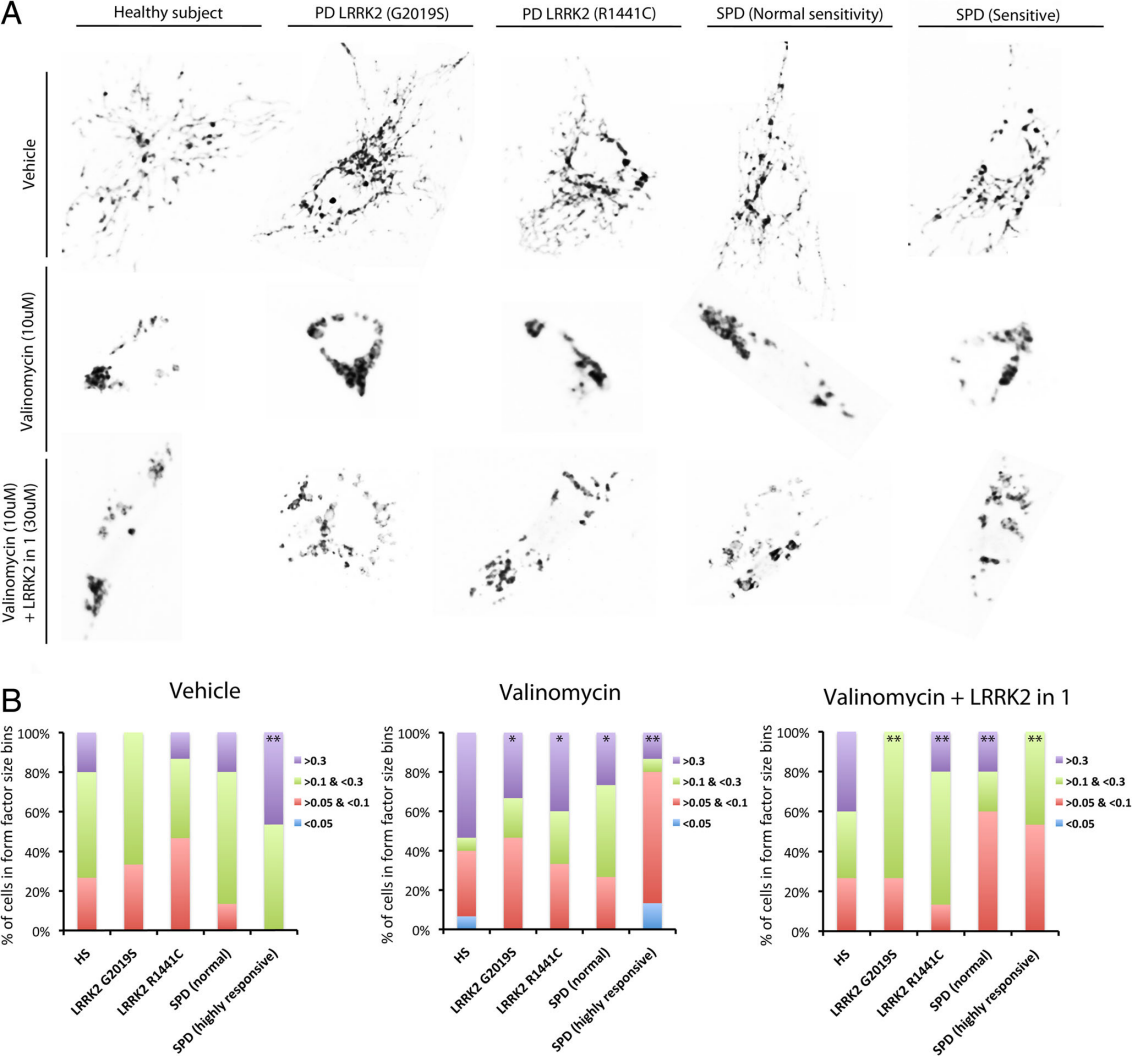
LRRK2-in-1 can restore the excessive mitochondrial network collapse caused by valinomycin in sporadic PD patient-derived fibroblast lines. Representation of mitochondrial networks in healthy subject controls, sporadic PD, and mutant LRRK2 (G2019S and R1441C) PD patient-derived fibroblast lines under vehicle conditions, valinomycin application, or co-application of valinomycin and LRRK2-in-1 for 24 h (**a**). Under vehicle conditions, both sensitive and non sensitive sporadic PD patient fibroblast lines had a more fragmented mitochondrial network as indicated by a higher average form factor, where a higher percentage of cells had a form factor score of over 0.3 (**b**). Following the application of valinomycin mitochondrial networks in sensitive sporadic PD-derived fibroblast lines showed an average decrease in form factor, with a higher percentage of cells scored less than 0.1 and more than 0.05 (**b**). In healthy subject control and mutant LRRK2 (G2019S and R1441C), PD patient-derived fibroblast lines the mitochondrial network was either collapsed or fragmented and each of the lines showing both high and low form factor scores. These scores were determined to be either above 0.3 or between 0.05 and 0.1 (**b**). LRRK2-in-1 treatment partially restored the collapse phenotype and form factor scores were normalized to between 0.1 and 0.3 (**b**). In less-sensitive sporadic PD patient-derived fibroblast lines restoration of form factor by LRRK2-in-1 was caused by an increase in form factor scores ranging between 0.05 and 0.1 (**b**). Graphs are annotated as *p < 0.05 and **p < 0.01 compared to healthy subject controls, as analyzed by a 1-way ANOVAs with Dunnett’s post hoc tests. Graphs are expressed at mean ± SEM. *N* = 4–5/group

In order to determine the degree of mitochondrial network connectivity and branching in PD patient-derived fibroblast lines, we used performed factor analysis on individual fibroblasts, as done previously for familial PD-derived fibroblast lines [18, 20]. We determined that under baseline conditions, the percent of sensitive sporadic PD patient-derived fibroblasts with a form factor of over 0.3 was significantly higher than HS controls (*p* < 0.01), (Fig. 2a, b), indicating a more fragmented mitochondrial network. In accordance, no sporadic PD patient-derived fibroblasts displayed a factor of lower than 0.05 in contrast to HS controls (Fig. 2a, b). Indicating that the mitochondrial network was less interconnected and more mitochondria were isolated. This is likely the effect of the increased fusion activating protein levels Mfn1 (*p* < 0.05) and OPA1 (*p* < 0.05), working synergistically with a decrease in the fission-activating protein Dlp1 (*p* < 0.05), (Supp. Fig. 5 - online resource). Sporadic PD patients that were highly sensitive to mitochondrial stress also had an increase of the mitochondrial fission protein MARCH5 at baseline (*p* < 0.05), (Supp. Fig. 5f - online resource). Upon valinomycin exposure, fibroblasts from both LRRK2 mutation carrying and sporadic PD lines displayed a significant decrease in form factor bins over 0.3 (G2019S = *p* < 0.05, R1441C = *p* < 0.05, normally sensitive SPD = *p* < 0.05 and highly sensitive sporadic PD lines = *p* < 0.01), (Fig. 2a, b), indicating a total collapse of mitochondria around the cell nucleus. The outer mitochondrial fusion protein Mfn2 was selectively increased in less-sensitive sporadic PD lines following valinomycin exposure (*p* < 0.05), yet was decreased in mutant LRRK2 G2019S (*p* < 0.05) and R1441C (*p* < 0.05) PD patient-derived fibroblast lines compared to healthy subject controls (Supp. Fig. 5c - online resource). The lack of mitochondrial connectivity and total collapse of mitochondria by valinomycin was also associated by decreased inner mitochondrial membrane fusion protein, OPA1, in all cell lines (*p* < 0.05), (Supp. Fig. 5d - online resource). The mitochondrial network collapse phenotype, was partially rescued by LRRK2 kinase inhibition in a non-cell line-dependent manner, as indicated by the reduction of form factor bins over 0.3 compared to valinomycin alone conditions (Fig. 2a, b). However, form factor bins of over 0.3 remained significantly lower in PD patient-derived fibroblast lines compared to HS controls (*p* < 0.01), (Fig. 2a, b). Although OPA1 levels were increased in healthy subject controls, mutant LRRK2 G2019S (*p* < 0.001), R1441C (*p* < 0.001) and less-sensitive sporadic lines (*p* < 0.001), following LRRK2 kinase inhibition, levels were not restored in more sensitive sporadic PD-derived fibroblast lines (Supp. Fig. 5d - online resource). Conversely, LRRK2-in-1 treatment was able to decrease the elevated levels of MARCH5 in all cell lines (*p* < 0.001), (Supp. Fig. 5f - online resource). After confirming the mitochondrial mislocalization and changes in fission and fusion dynamics in sporadic PD patient-derived fibroblast lines, we next analyzed the activity of the mitochondria.

**Fig. 3:**
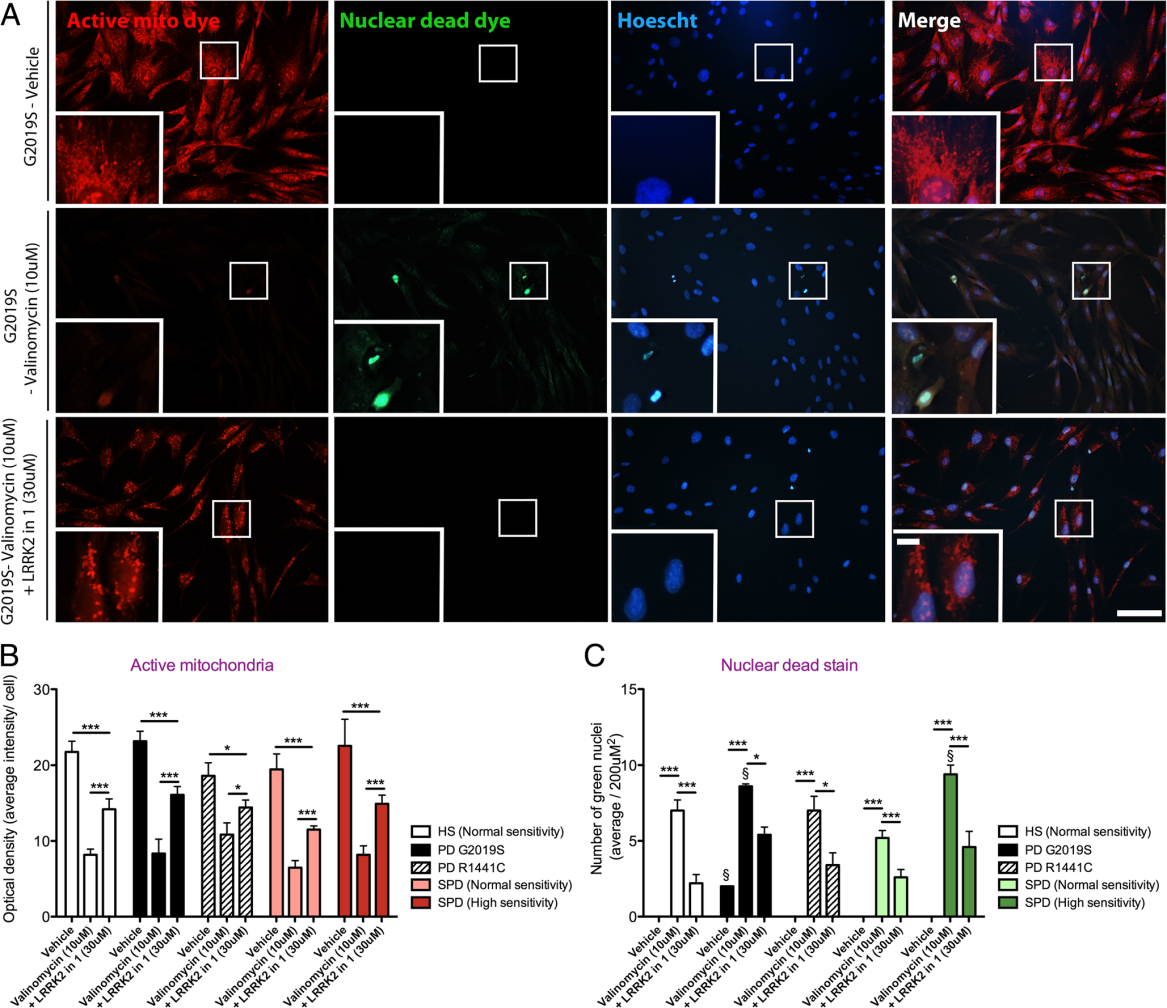
LRRK2-in-1 restores mitochondrial membrane potential loss caused by valinomycin exposure. The active mitochondria dye (*red*) and dead nuclear dye (*green*) was used as an indicator of the loss of mitochondrial membrane potential and cell death, respectively, caused by 24 h of valinomycin (10 μM) exposure (**a**). Co-treatment with LRRK2-in-1 for the full 24 h rescued the loss of mitochondrial membrane potential and cell death (**a**). There was no significant difference in the intensity of mitochondrial membrane potential sensing dye between fibroblast lines at baseline (**b**). Valinomycin exposure reduced the intensity of the mitochondrial membrane potential sensing dye, in all fibroblast lines, which could be partially restored by LRRK2-in-1, (**b**). Under vehicle conditions, dead permeated nuclei were only observed in mutant LRRK2 (G2019S) fibroblast lines (**c**). The application of valinomycin caused an increase in the number of dead permeated nuclei in all cell lines, yet were preferentially higher in mutant LRRK2 (G2019S) and sensitive sporadic PD patient-derived fibroblast lines (**c**). LRRK2-in-1 reduced the presence of dead permeated nuclei in all cell lines (**c**). Graphs are annotated as ^§^ *p* < 0.05 compared to healthy subject controls and *p < 0.05 and ***p < 0.001 compared to vehicle conditions, as analyzed by a multivariate ANOVAs with Dunnett’s post hoc tests. Graphs are expressed at mean ± SEM. *N* = 4–5/group. Low-magnification *scale bar* = 200 μM and high-magnification *scale bar* = 50 μM

In contrast to the mitochondrial mislocalization phenotypes of sporadic PD patient-derived fibroblast lines at baseline, mitochondria remained active, when analyzed by a live active mitochondrial specific dye (Fig. 3a, b). Twenty-four hours following the application of valinomycin, the fluorescent intensity of the mitochondria was decreased to negligible levels in all fibroblast lines (*p* < 0.001), (Fig. 3a, b). There was no significant difference in the fluorescent intensity of the active mitochondria dye between mutant LRRK2 (G2019S and R1441C), sporadic groups and healthy subject controls under vehicle conditions (Fig. 3a, b). The co-application of valinomycin and LRRK2-in-1 partially restored the activity of the mitochondria (*p* < 0.001), which was also independent of cell line (Fig. 3a, b). The number of dead cells was analyzed simultaneously using a nuclear dead stain, indicating rupture of the nuclear membrane. Valinomycin increased the number permeated nuclei in all cell lines (*p* < 0.001), (Fig. 3c). Dead cells were also preferentially increased in mutant LRRK2 (G2019S) (*p* < 0.05) and highly sensitive sporadic PD lines (*p* < 0.05), verifying results from the LDH assay. Coinciding with the rescue of mitochondrial membrane potential, LRRK2-in-1 also reduced the number of ruptured nuclear membranes in all cell lines and the preferential increase in ruptured nuclei observed in sensitive sporadic PD (*p* < 0.001) and mutant LRRK2 (G2019S) (*p* < 0.05) derived fibroblast lines (Fig. 3c).

Given this cell death profile and our finding that valinomycin induces oxidative stress, we analyzed protein levels of the oxidative stress sensitive mitochondrial complexes, which could potentially be used as a reportable biomarker of cellular function. LRRK2 (G2019S) mutation carrying lines had elevated levels of mitochondrial complexes I (*p* < 0.05), II (*p* < 0.05), III (*p* < 0.05), and V (*p* < 0.05), compared to healthy subject control lines, which was not exacerbated further by valinomycin treatment (Supp. Fig. 6 - online resource). However, treatment by LRRK2-in-1 specifically reduced protein levels of mitochondrial complexes I (*p* < 0.05) and III (*p* < 0.001) to that of healthy subject controls (Supp. Fig. 6- online resource). In contrast, less-sensitive sporadic PD lines and healthy subject controls had heightened levels of mitochondrial complexes II (*p* < 0.001) and III (*p* < 0.001) following LRRK2-in-1 treatment, which was not observed in highly sensitive sporadic PD patient-derived fibroblast lines (Supp. Fig. 6 - online resource). This indicates a divergent mitochondrial complex level correction pathway, initiated by kinase inhibition, between familial and sporadic PD fibroblast samples (Supp. Fig. 6 - online resource).

### LRRK2-in-1 Treatment Regulates PINK1 and Parkin, and Mitochondrial and Lysosome Co-localization in PD Patient-Derived Fibroblast Lines

**Fig. 4:**
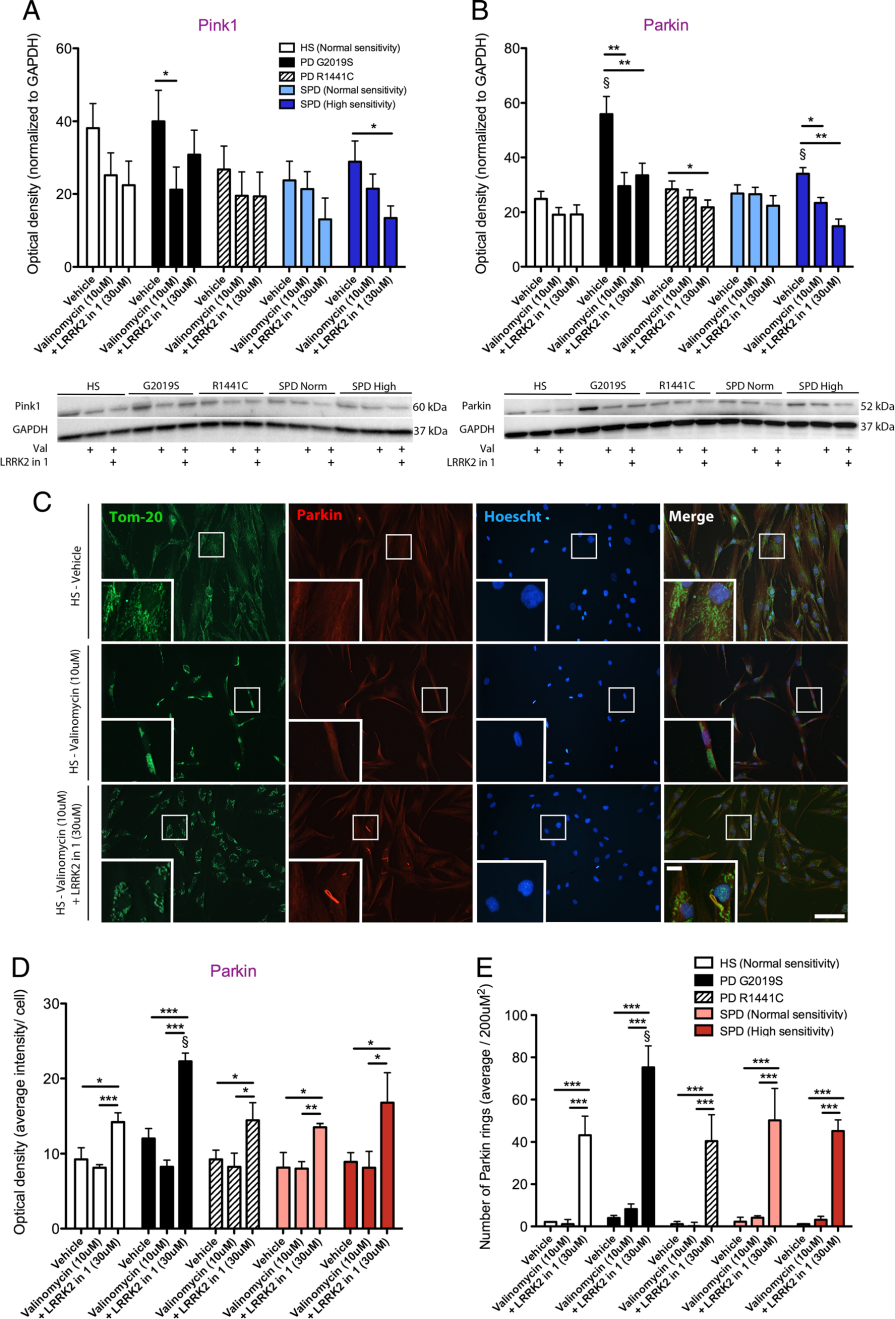
LRRK2-in-1 reduces PINK1 and Parkin levels in sporadic PD patient fibroblasts and is associated with the sequestering of Parkin into ring formations. PINK1 protein levels were significantly increased in mutant LRRK2 (G2019S) PD patient-derived fibroblast lines with vehicle treatment compared to valinomycin treatment, given for 24 h (**a**). PINK1 protein levels were also increased in sensitive sporadic PD patient-derived fibroblast lines with vehicle treatment compared to LRRK2-in-1 treatment (**a**). The protein levels of Parkin were significantly elevated under vehicle conditions in mutant LRRK2 (G2019S) and sensitive sporadic PD patient-derived fibroblast lines, compared to both valinomycin treatment alone and valinomycin and LRRK2-in-1 co-treatment (**b**). The location of Parkin (*red*) was found to be evenly distributed throughout the cytoplasm of fibroblast lines under vehicle and valinomycin treatment conditions, yet was sequestered in to rings with LRRK2-in-1 and valinomycin co-treatment (**c**). The mean intensity of Parkin did not differ between vehicle and valinomycin treatment, yet was increased following valinomycin and LRRK2-in-1 co-treatment in all fibroblast cell lines (**d**). There were negligible levels of Parkin rings present in fibroblast lines under both vehicle and valinomycin conditions (**a**, **e**). Following the application of LRRK2-in-1, the number of cells with Parkin rings was significantly increased in all fibroblast lines and were preferentially increased in mutant LRRK2 (G2019S) and sporadic PD patient-derived fibroblast lines (**a**, **e**). Parkin rings were partially colocalized with the mitochondria (**a**). Graphs are annotated as ^§^ *p* < 0.05 compared to healthy subject controls and *p < 0.05, **p < 0.05, and ***p < 0.001 compared to vehicle conditions, as analyzed by a multivariate ANOVAs with Dunnett’s post hoc tests. Graphs are expressed at mean ± SEM. *N* = 3–5/group. Low-magnification *scale bar* = 200 μM and high-magnification *scale bar* = 50 μM

PINK1 and Parkin initiate the mitophagy pathway for the removal of damaged mitochondria [35, 36]. We show that deficits in mitochondrial and lysosome co-localization, indicative of altered mitophagy, and changes in the expression of PINK1 and Parkin can be detected in sporadic PD patient fibroblast lines. We further show that LRRK2-in-1 regulates levels of mitochondrial and lysosome co-localization. A deficit in mitophagy is an important reportable phenotype to measure in PD patient samples as increased lysosomal activity has been implicated in the neurite shortening phenotype of iPSC-derived dopamine neurons harboring mutant LRRK2 (G2019S) [14]. Full-length PINK1 protein levels were decreased G2019S and sporadic PD patient-derived fibroblasts lines following valinomycin exposure (*p* < 0.05), which was not rescued by the application of LRRK2-in-1 (Fig. 4a). The full-length form of PINK1 is proteolytically processed and cleaved upon entry into mitochondria, in a voltage-dependent manner. The levels of cleaved PINK1 did not change, in any lines, following valinomycin exposure, yet were significantly increased in mutant LRRK2 G2019S fibroblasts in all treatment conditions, *p* < 0.05, (Supp. Fig. 7a - online resource). Parkin protein levels were elevated in sporadic PD patient-derived fibroblast lines that were highly sensitive to mitochondrial stress (*p* < 0.05) and LRRK2 (G2019S) mutation carrying lines (*p* < 0.05) at baseline (Fig. 4b). The application of valinomycin reduced heightened levels to that of healthy subject control lines (*p* < 0.05) and the co-application of LRRK2-in-1 had not further effect (Fig. 4b). This finding was also verified using a second Parkin antibody recognizing a different epitope of the protein (Supp. Fig. 7b - online resource). Parkin is distributed evenly throughout the cytoplasm under baseline conditions and following valinomycin treatment (Fig. 4c). However, the co-administration of valinomycin and LRRK2-in-1 causes Parkin to be sequestered into structures we have termed “Parkin rings” (Fig. 4c). Parkin rings were partially colocalized to mitochondria (Fig. 4c). The application of LRRK2-in-1 alone does not cause the formation of Parkin rings, indicating mitochondria must first be under conditions of stress (Supp. Fig. 8b - online resource). The fluorescent intensity of the Parkin staining did not differ between fibroblast lines at baseline and did not increase with valinomycin treatment (Fig. 4d). Conversely, the fluorescent intensity of Parkin was increased by the co-administration of valinomycin and LRRK2-in-1 in all cell lines (*p* < 0.001), (Fig. 4d). This is directly related to the accumulation of Parkin, as the number of cells that contain Parkin rings was also increased following the co-administration of valinomycin and LRRK2-in-1 (*p* < 0.001), (Fig. 4e). PD patient-derived fibroblast lines harboring a LRRK2 mutation (G2019S) had significantly more Parkin rings (*p* < 0.05), compared to healthy subject controls and the other PD patient-derived fibroblast lines (Fig. 4e).

**Fig. 5:**
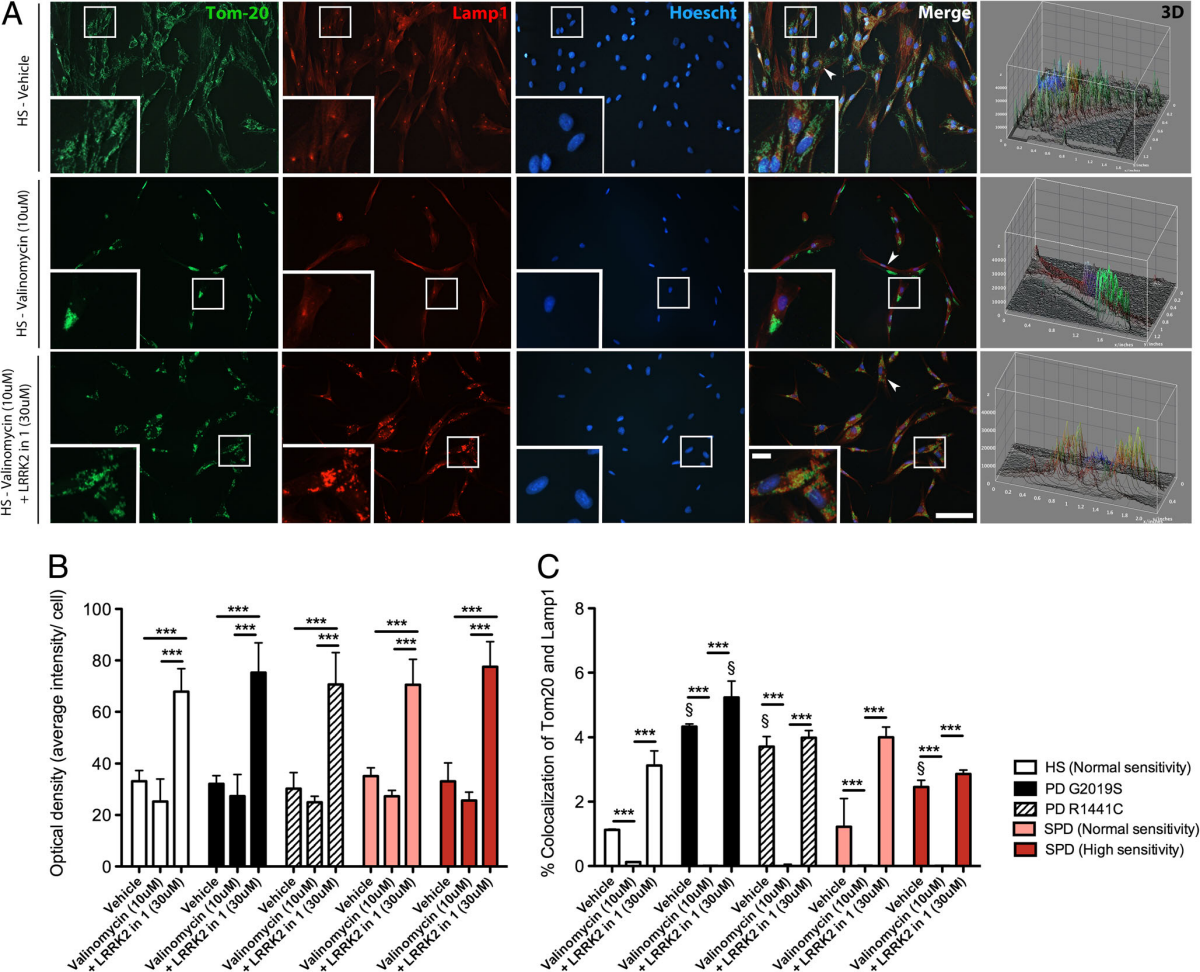
LRRK2-in-1 induces mitochondrial localization to the lysosome that is preferentially increased in mutant LRRK2 and a subpopulation of sporadic PD patient-derived fibroblast lines. Under vehicle and valinomycin conditions the subcellular localization of LAMP1-positive lysosomes (*red*) in fibroblasts lines is confined to the perinuclear compartment (**a**). Under vehicle conditions, a small number of Tom20-positive mitochondria (*green*) could be observed in the lysosome in all cell lines, yet the application of valinomycin for 24 h prevented LAMP1 and Tom20 co-localization (**a**). LRRK2-in-1 and valinomycin co-treatment for 24 h caused an increase in lysosome formation throughout the cytoplasm and LAMP1-positive vesicles and Tom20-positive mitochondria were partially colocalized (**a**). The intensity of LAMP1 was increased in all fibroblast lines following the co-application of valinomycin and LRRK2-in-1 (**b**). The percent of mitochondria in the lysosome, under vehicle conditions, was highest in mutant LRRK2 (G2019S) and highly sensitive sporadic PD patient-derived fibroblast lines (**c**). The percent co-localization of mitochondria and lysosomes was decreased to negligible levels in all lines following valinomycin exposure (**c**). Co-application of valinomycin and LRRK2-in-1 caused a restoration of mitochondria and lysosome co-localization in mutant LRRK2 (G2019S and R1441C) and highly sensitive PD patient-derived fibroblast cell lines, which were comparable to levels observed at baseline (**c**). In less-sensitive sporadic PD patient and healthy subject control-derived fibroblast lines, mitochondria and lysosome co-localization was increased above baseline levels following LRRK2-in-1 treatment (**c**). Graphs are annotated as ^§^ *p* < 0.05 compared to healthy subject controls and ***p < 0.001 compared to vehicle conditions, as analyzed by a multivariate ANOVAs with Dunnett’s post hoc tests. Graphs are expressed at mean ± SEM. *N* = 4–5/group. Low-magnification *scale bar* = 200 μM and high-magnification *scale bar* = 50 μM

Given the changes in PINK1 and Parkin, we next measured the degree of mitophagy in fibroblast cell lines, which can be measured by the co-fluorescent staining of mitochondria using Tom20 and lysosomes using LAMP1. This was determined using a defined co-localization algorithm, which masks and calculates the percentage of overlap between Tom20 and LAMP1 stained areas in comparison to the total Tom20 stained area (Supp. Fig. 9). At baseline and following valinomycin treatment, all cell lines displayed a single lysosome structure in the perinuclear compartment (Fig. 5a). Accordingly, the fluorescent intensity of the lysosome staining was not significantly different between vehicle and valinomycin-treated fibroblast lines (Fig. 5b). The co-administration of valinomycin and LRRK2-in-1 caused an increase in the number and size of LAMP1-positive vesicles, which were present throughout the cytoplasm (Fig. 5a). Consequently, the fluorescent intensity of LAMP1-positive lysosomes, after co-treatment was increased twofold in all cell lines (*p* < 0.001), (Fig. 5b). Despite the comparable LAMP1 changes of healthy subject control and PD patient-derived fibroblasts, mitophagy levels were differently altered (Fig. 5c). At baseline, there was more co-localization of mitochondria and lysosomes in mutant LRRK2 G2019S (*p* < 0.05) and R1441C (*p* < 0.05) and highly sensitive sporadic PD (*p* < 0.05) compared to both healthy subject controls and less-sensitive sporadic PD fibroblast lines (Fig. 5c). Mitochondrial co-localization to the lysosome was also confined to the perinuclear compartment in all cell lines under vehicle conditions (Fig. 5a). Tom20 and LAMP1 co-localization was not observed in any cell lines following valinomycin treatment (Fig. 5a, c), indicating a severe defect in the removal of damaged mitochondria. The co-localization of Tom20 and LAMP1 was restored to baseline levels following the co-application of valinomycin and LRRK2-in-1 in mutant LRRK2 G2019S (*p* < 0.001) and R1441C (*p* < 0.001) PD and highly sensitive sporadic PD lines (*p* < 0.001), (Fig. 5a, c). In contrast, mitophagy levels were above baseline levels in less-sensitive sporadic PD lines (*p* < 0.001) and healthy subject controls (*p* < 0.001) with the addition of LRRK2-in-1 (Fig. 5a, c). The regulation of mitophagy by LRRK2-in-1 also indicates another potential therapeutic benefit of this compound administration. Despite the increase of lysosome size and number with the application of LRRK2-in-1 alone, the co-localization of Tom20 and LAMP1 was not increased above baseline levels (Supp. Fig. 8a - online resource). This indicates that the presence of damaged mitochondria is required for increased mitophagy, and not simply aberrant lysosome formation.

## Discussion

### Sporadic PD Patient-Derived Fibroblast Biomarkers

It is an important goal to determine reportable biomarker phenotypes for use as preclinical diagnostic tools for sporadic PD. Fibroblasts have previously been characterized in cases of familial PD, and we and others have determined robust mitochondrial phenotypes from lines harboring LRRK2 [9–13], PINK1 [10, 15], and Parkin [16–20] mutations, both at baseline and under conditions of pharmacological stress. Therefore, in this series of experiments, we have investigated whether sporadic *(non-familial)* PD patient-derived fibroblast lines also show mitochondrial phenotypes and compare them against lines derived from LRRK2 mutation (G2019S and R1441C) carrying PD patients. In general, we find that mitochondrial-associated phenotypes are greater in fibroblasts harboring a G2019S mutation in comparison to those carrying an R1441C mutation. This is in keeping with the fact that LRRK2 kinase activity is preferentially increased by the G2019S mutation, compared to other mutant forms [37, 38]. We further show that phosphorylation at S955 was exclusivity detected in fibroblast lines carrying the G2019S mutation and may therefore this site may be of importance to the increased kinase activity observed in previous studies.

A key finding from our data sets show that sporadic lines can be divided into two statistically distinct groups based on phenotypic analysis of a number of reportable assays. Approximately 50 % of the sporadic PD patient lines tested lacked significant phenotypes when stressed using the mitochondrial specific toxin valinomycin, yet the remaining sporadic PD patient-derived cell lines were highly sensitive to mitochondrial stress. Sensitive sporadic PD-derived fibroblast lines had comparable mitochondrial phenotypes to LRRK2 mutation carrying lines. Although a sub-classification of sporadic cells lines has not previously been attempted, this may have potential importance for recruiting patients for clinical trial, as not all sporadic PD cases may respond the same way to novel treatments. Secondly, these samples provide an accessible pharmacodynamic system for testing individual drugs.

Specifically, we determined that sporadic PD patient-derived fibroblast derived fibroblast lines that displayed a more fragmented mitochondrial network, increased levels of nitric oxide, elevated PINK1/Parkin levels, and heightened mitophagy at baseline; were also highly sensitive to valinomycin toxicity. These findings represent the first instance where a detectable increase in mitophagy was observed in sporadic PD patient samples and hence treatments that regulate mitochondrial turnover may represent a unified therapeutic approach for PD. It has been reported that mutant LRRK2 (R1441C and G2019S) expression can induce mitophagy in vitro, causing the removal of mitochondria in neurons, resulting in dendrite and neurite shortening [14, 39, 40]. We now show increased mitochondria and lysosome co-localization, implying heightened mitophagy, can also be observed in human fibroblasts lines harboring LRRK2 mutations, at baseline, emphasizing the use of skin biopsy-derived fibroblasts as a candidate tool to determine biomarker phenotypes for neuronal dysfunction. Moreover, it is possible that aberrant mitophagy in a cohort of sporadic PD patients may be related to early dopamine neuron terminal loss in the disease. These data also suggest that mitochondria and lysosome co-localization, in sporadic PD and mutant LRRK2 mutation carriers is likely to be the downstream consequence of increased Parkin protein levels. Elevated Parkin levels have previously been observed in sporadic PD fibroblast cells and this was correlated with increased protein ubiquitination [26]. Despite the fact that mitochondria and lysosome co-localization was increased at baseline in sporadic PD and mutant LRRK2 fibroblast lines, this is reduced to negligible levels following a 24-h exposure of valinomycin at 10 μM. We suggest that this was caused by the lack of Parkin translocation to the mitochondria, which is thought to be prerequisite to mitophagy [36]. In contrast, Parkin relocalization to the mitochondria was previously observed following the administration valinomycin at a 1-μM concentration, at 12-h post exposure [19]. Therefore, this phenomenon is likely time dependent and may not occur with prolonged mitochondrial stress.

A significant loss of Parkin and FL PINK1 protein levels was observed in sensitive sporadic PD fibroblast lines and those carrying a G2019S LRRK2 mutation following valinomycin exposure for 24 h. It has previously been determined that Parkin levels are decreased upon valinomycin exposure in non-PD cell types [19]; however, this was not accompanied by a loss of PINK1 [19]. We therefore suggest that sensitive sporadic PD fibroblast lines and those carrying a G2019S mutation may therefore have alteration in the processing of FL PINK1 at later time points with sustained mitochondrial stress.

Although the majority of cellular sporadic PD phenotypes recapitulated those of LRRK2 mutation carrying lines, the mitochondrial fission protein Dlp1 was increased in LRRK2 mutation carrying lines, yet decreased in sporadic PD lines. This may have implications for the newly developed discovery approaches aimed to knock down Dlp1 for use in PD [41]. Based on our observations we suggest that Dlp1 knockdown would be most suited to mutant LRRK2 (R1441C and G2019S) carrying PD patients. Conversely, the loss of Dlp1 was recently found to cause the abnormal movement of mitochondria and the degradation of dopamine neurons [42]; therefore, it is also possible that loss of Dlp1 may be a biomarker for neuronal demise.

Following the application of valinomycin, highly sensitive sporadic PD patient-derived fibroblast lines displayed an increased collapse phenotype of mitochondria around the nucleus, which occurred at a faster rate than in healthy subject control cells. We have found that this cellular phenotype is directly relevant to dopamine neurons, in vivo, as mitochondrial collapse is seen in the remaining dopamine neurons in post mortem tissue samples [29]. We suggest that this phenotype was caused by the increase in the mitochondrial fusions proteins Opa1 and Mfn2 and/ or the decrease in the mitochondrial fission protein Dlp1. These changes likely contribute to mitochondrial collapse to the perinuclear compartment during mitochondrial stress. Conversely, the loss of Opa1 and increase of Dlp1 has also been found in other in mutant Parkin PD patient-derived fibroblast lines [43] and this may represent mitochondrial fragmentation, which occurs before total mitochondrial collapse. The sensitive group of sporadic PD lines displayed heightened superoxide levels, loss of mitochondrial membrane potential, a cessation of mitochondria and lysosome co-localization and ultimately a preferential increase in toxicity. These phenotypes recapitulated the mutant LRRK2 cellar phenotypes.

We propose that the divergent phenotypic responses observed between groups of sporadic PD patient samples may be useful in preclinical diagnostics, where it may be possible to discern patients that respond to novel therapeutics and those who do not. In contrast to the valinomycin application, sporadic PD patient fibroblast samples treated with the complex I inhibitor rotenone showed a decreased mitochondrial respiration but did not display changes in autophagy or levels of reactive oxygen species [26]. It may therefore be possible to distinguish pathogenic mechanisms in sporadic PD using different pharmacological stressors.

### Sporadic PD Patient-Derived Fibroblast Lines Respond to LRRK2 Kinase Inhibition

It is currently unknown whether LRRK2 kinase inhibition, using small molecules, would be beneficial in sporadic PD cases. Since we have defined specific phenotypes in fibroblasts derived from sporadic PD patients, we therefore explored whether the inhibition of LRRK2 kinase activity, a current potential therapeutic intervention, would reduce the phenotypes observed in sporadic PD patient-derived fibroblast lines. These assays could also be used as an initial screening process for selecting sporadic PD patients for clinical trials.

Elevated levels of pathogenic pLRRK2 (S935) and (S973) species were increased in sensitive sporadic PD patient-derived fibroblast lines under conditions of mitochondrial stress. Application of LRRK2-in-1 also decreased pLRRK2 levels to baseline and further rescued the loss of mitochondrial membrane potential. These data suggest that LRRK2-in-1 treatment may therefore be of value in a subgroup of sporadic PD patients in addition to LRRK2 mutation carriers.

Our findings indicate that the concentration of LRRK2-in-1 required for efficient rescue of mitochondrial-related changes in human dermal fibroblasts is higher than used to mediate changes other neuronal cell types. A dose of 0.5 μM was used to attenuate the toxicity caused by mutant LRRK2 transfected SH-SY5Y cells [32], whereas a dose of 3 μM was required for efficient LRRK2 kinase inhibition in transfected HEK293 cells [44]. In contrast, a dose of 5 μM was needed for application to primary cortical neurons [45]. Therefore, dose-response curves are critically important to determine the minimal dose required for each experimental paradigm to avoid potential “off target” effects. Although there are multiple kinase inhibitors available that target mutant forms of LRRK2, the search for new potent WT LRRK2 kinase inhibitors is much needed for future studies investigating LRRK2 phosphorylation in sporadic PD. The recently developed LRRK2 kinase inhibitor 3-[4-(morpholin-4-yl)-7H-pyrrolo[2,3-d]pyrimidin-5-yl]benzonitrile (PF-06447475) holds promise for the treatment of sporadic PD since it has a high affinity to target WT LRRK2 and has been validated for use in vivo [46].

LRRK2-in-1 was previously found to enhance the conversion LC3 I to LC3 II which is required for the formation of the autophagosome [34]. Mitophagy levels, as inferred by mitochondrial and lysosome co-localization, were also restored to baseline levels, following LRRK2 kinase inhibition, in LRRK2 mutation carrying and sensitive sporadic lines. Conversely, the level of mitochondrial and lysosome co-localization was increased above baseline levels in healthy subject controls and less valinomycin sensitive sporadic lines. These data support the notion that mitophagy could be potentially used as a biomarker in fibroblasts. It is also reasonable that other organelles such as the endoplasmic reticulum and peroxisomes may also be degraded by macroautophagy in fibroblasts derived from PD patients. Hence, it is possible other disease-relevant biomarkers may be found in future experiments.

We find that restoration or increased mitochondrial and lysosome co-localization was not dependent on increased levels of PINK1 and Parkin but rather concurrent with the formation of Parkin into circular aggregated structures, which we have termed “Parkin rings,” that partially co-localize with mitochondria. Although the formation of these structures has not previously been identified, it is likely that their formation is a direct consequence of LRRK2 kinase inhibition. LRRK2 functionally interacts with the C-terminal R2 RING finger domain of Parkin and Parkin interacts with the COR domain of LRRK2 [47]. Further, the over expression of Parkin is protective against mutant LRRK2 (G2019S) mediated degeneration [48]. We therefore note the accumulation of Parkin rings in close proximity to mitochondria, under conditions of LRRK2 kinase inhibition, is a means of locally increasing Parkin for the stimulation of mitophagy, or may not play a direct role. The aggregation of Parkin has previously been reported in healthy subject control iPSC-derived neurons but not PINK1 mutant lines following the application of valinomycin [49]. Unlike Parkin ring formation, the aggregation pattern previously found was present throughout the soma and this was correlated with the absence of mitophagy [49]. Therefore the sequestering of Parkin may have dual roles in both the inhibition and stimulation of mitophagy, dependent on the cellular stress phenotype.

## Summary

A group of sporadic PD patient’s fibroblasts showed specific mitochondrial phenotypes at baseline and when stressed, equivalent to mutant LRRK2 mutation carrying PD patients. We suggest that these defined PD-associated phenotypes can be used as a biomarker for mitochondrial dysfunction based on a number of reportable assays. We further conclude that sporadic PD patient fibroblasts respond to LRRK2 kinase inhibition and treatment with such a compound was able to rescue mitochondrial membrane potential and restore/ increase mitochondrial and lysosome co-localization. This fibroblast phenotype biomarker screen suggests that some sporadic PD patients may be suitable candidates for LRRK2 kinase inhibition treatment in clinical trials. It will also be important in future experiments to test whether PD-related mitochondrial phenotypes observed in fibroblasts are also seen in cells that can be isolated from a blood sample. This would enable a faster screening procedure that could potentially be more easily utilized in the clinic. It was also recently shown that familial PD-derived fibroblast lines can be used for large scale drug screens to identify novel compounds [21] and we suggest sporadic fibroblast lines may also be suitable candidates for such screens.

## Electronic supplementary material

Below is the link to the electronic supplementary material.

ESM 1 (PDF 17096 kb)
