# Supplementary material for: Fibroblast Biomarkers of Sporadic Parkinson’s Disease and LRRK2 Kinase Inhibition
Source: Mol Neurobiol. 2015 Sep 23;53(8):5161–77. doi: 10.1007/s12035-015-9435-4 (PMC5012155; doi:10.1007/s12035-015-9435-4)
Supplement: Supplementary file 1 — (PDF 17096 kb) [file 12035_2015_9435_MOESM1_ESM.pdf]

*Molecular Neurobiology*

# Supplementary Material

**Fibroblast biomarkers of sporadic Parkinson's disease and LRRK2 kinase inhibition**

G. A. Smith[1†], J. Jansson[1†], E. M. Rocha[1], T. Osborn[1], P. J. Hallett[1], O. Isacson[1]*

[1]McLean Hospital/ Harvard Medical School, Neuroregeneration Research Institute, Center for Neuroregeneration Research, Belmont, 02478, U.S.A

[†] These authors contributed equally

* Correspondence:

Ole Isacson, MD-PhD
Neuroregeneration Research Institute
McLean Hospital/Harvard Medical School
115 Mill Street
Belmont, MA 02478 USA
isacson@hms.harvard.edu
Tel: (617) 855-3283

**Supplementary Table 1. Identification numbers and information on fibroblast lines used.**

| Catalog ID | Description | Sex | Biopsy Age | Age of disease Onset |
|---|---|---|---|---|
| ND30159 | Sporadic PD[1] | Female | 76 | 72 |
| ND32157 | Sporadic PD[2] | Female | 65 | 42 |
| ND32462 | Sporadic PD[2] | Male | 75 | 75 |
| ND32697 | Sporadic PD[2] | Male | 58 | 55 |
| ND34265 | Sporadic PD[2] | Male | 62 | 55 |
| ND35302 | Sporadic PD[1] | Male | 69 | 58 |
| ND35976 | Sporadic PD[1] | Male | 63 | 59 |
| ND39538 | Sporadic PD[1] | Female | 72 | 61 |
| ND38528 | Sporadic PD[2] | Female | 65 | 61 |
| ND39999 | Sporadic PD[1] | Male | 63 | 41 |
| AG06010 | Control | Female | 62 | n/a |
| AG05278 | Control | Female | 65 | n/a |
| AG07141 | Control | Male | 66 | n/a |
| AG11489 | Control | Male | 66 | n/a |
| AG05265 | Control | Female | 61 | n/a |
| AG11743 | Control | Female | 76 | n/a |
| AG06959 | Control | Male | 67 | n/a |
| AG06241 | Control | Male | 61 | n/a |
| AG04061 | Control | Male | 66 | n/a |
| AG04454 | Control | Male | 66 | n/a |
| AG04461 | Control | Male | 66 | n/a |
| AG13220 | Control | Male | 66 | n/a |
| AG04355 | Control | Male | 67 | n/a |
| AG06281 | Control | Male | 67 | n/a |
| sc1007 | PD (LRRK2 G2019S) | unknown | unknown | Unknown |
| ND92492 | PD (LRRK2 G2019S) | Male | 72 | 58 |
| PI-1353 C5 | PD (LRRK2 G2019S) | unknown | unknown | Unknown |
| PI-1679 C19 | PD (LRRK2 G2019S) | unknown | unknown | Unknown |
| MSC023 D-113 | PD (LRRK2 R1441C) | Male | 61 | Unknown |
| MSC029 D-121 | PD (LRRK2 R1441C) | Female | 72 | Unknown |
| MSC030 D-122 | PD (LRRK2 R1441C) | Female | 66 | Unaffected |
| MSC031 D-139 | PD (LRRK2 R1441C) | Female | 41 | Unaffected |

[1]Sporadic PD fibroblast lines classified as 'normally sensitive' to valinomycin.

[2]Sporadic PD fibroblast lines classified as 'highly sensitive' to valinomycin.

**Supplementary Fig. 1**

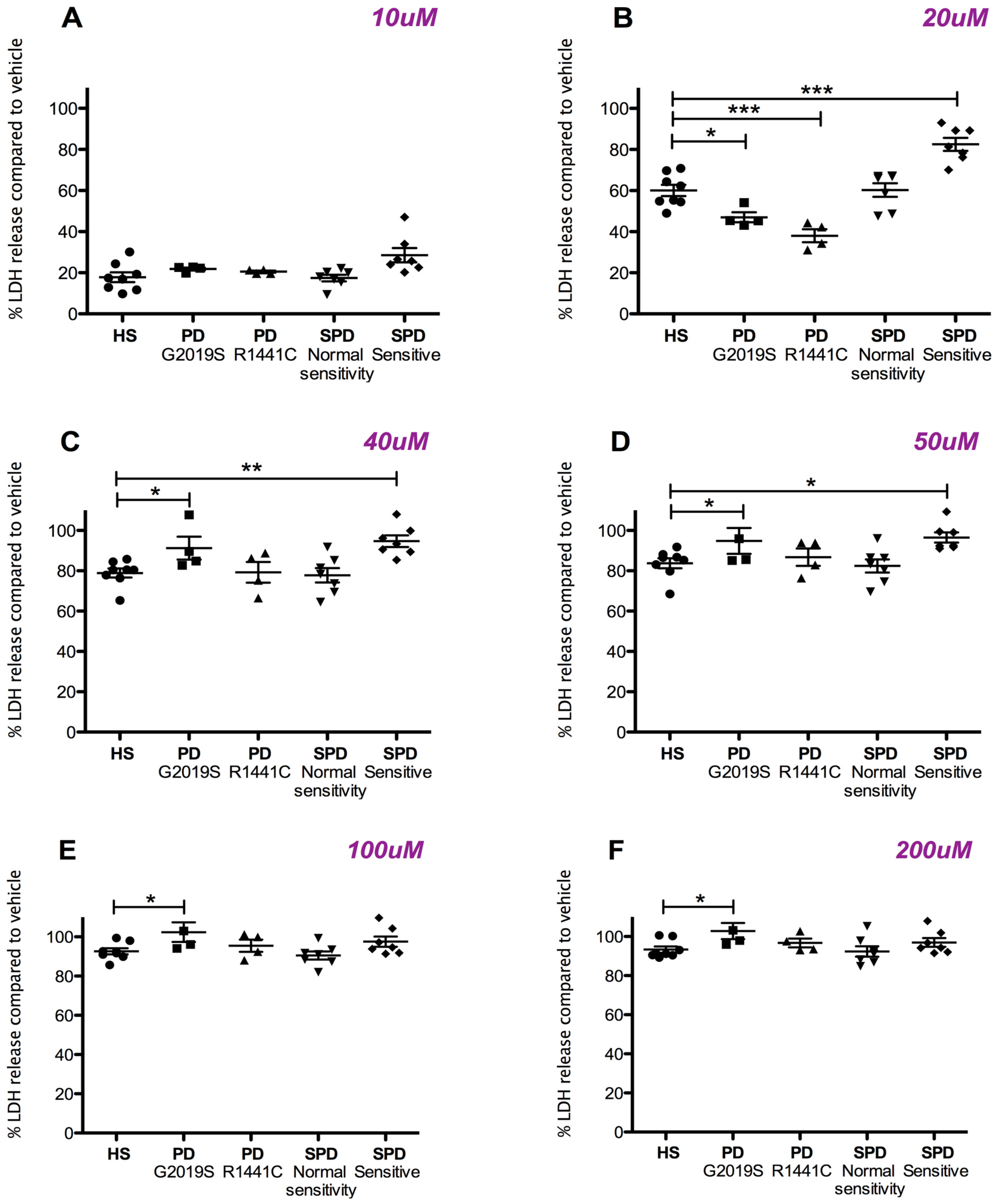

**Supplementary Fig. 1. PD patient fibroblast line vulnerability profile to the mitochondrial stressor valinomycin reveals two distinct populations of sporadic PD patient fibroblast lines.** Fibroblast cell lines derived from healthy subject controls, mutant LRRK2 (G2019S and R1441C) and sporadic PD patients showed increased vulnerability to escalating doses of valinomycin, given for 24 hrs, as analyzed by a LDH release assay. Specifically, two defined groups of sporadic PD patients could be observed based on their valinomcyin toxicity response and were classified as 'sensitive' or 'normally sensitivity' compared to healthy subject control derived lines. Minimal toxicity was observed at 10µM of valinomcyin and was equivalent between groups (A). At 20µM of valinomycin mutant LRRK2 (G2019S and R1441C) PD patient derived fibroblast lines showed a reduced toxicity profile compared to healthy subject controls, whereas sensitive sporadic PD fibroblast lines displayed an increase in toxicity (B). A preferential increase in cell death could be observed in mutant LRRK2 (G2019S) and sensitive sporadic PD patient derived fibroblast lines at valinomcyin doses of 40 and 50µM (C-D). Valinomycin doses of 100 and 200µM caused server toxicity in all cell lines, with exacerbated toxicity evident in the mutant LRRK2 (G2019S) PD patient derived fibroblast lines (E-F). Graphs are annotated as $^{*}p<0.05$, $^{**}p<0.01$ and $^{***}p<0.001$ compared to healthy subject controls, as analyzed by a 1-way ANOVAs with Dunnett's *post hoc* tests. Graphs are expressed at mean ± SEM. N=4-14/ group.

**Supplementary Fig. 2.**

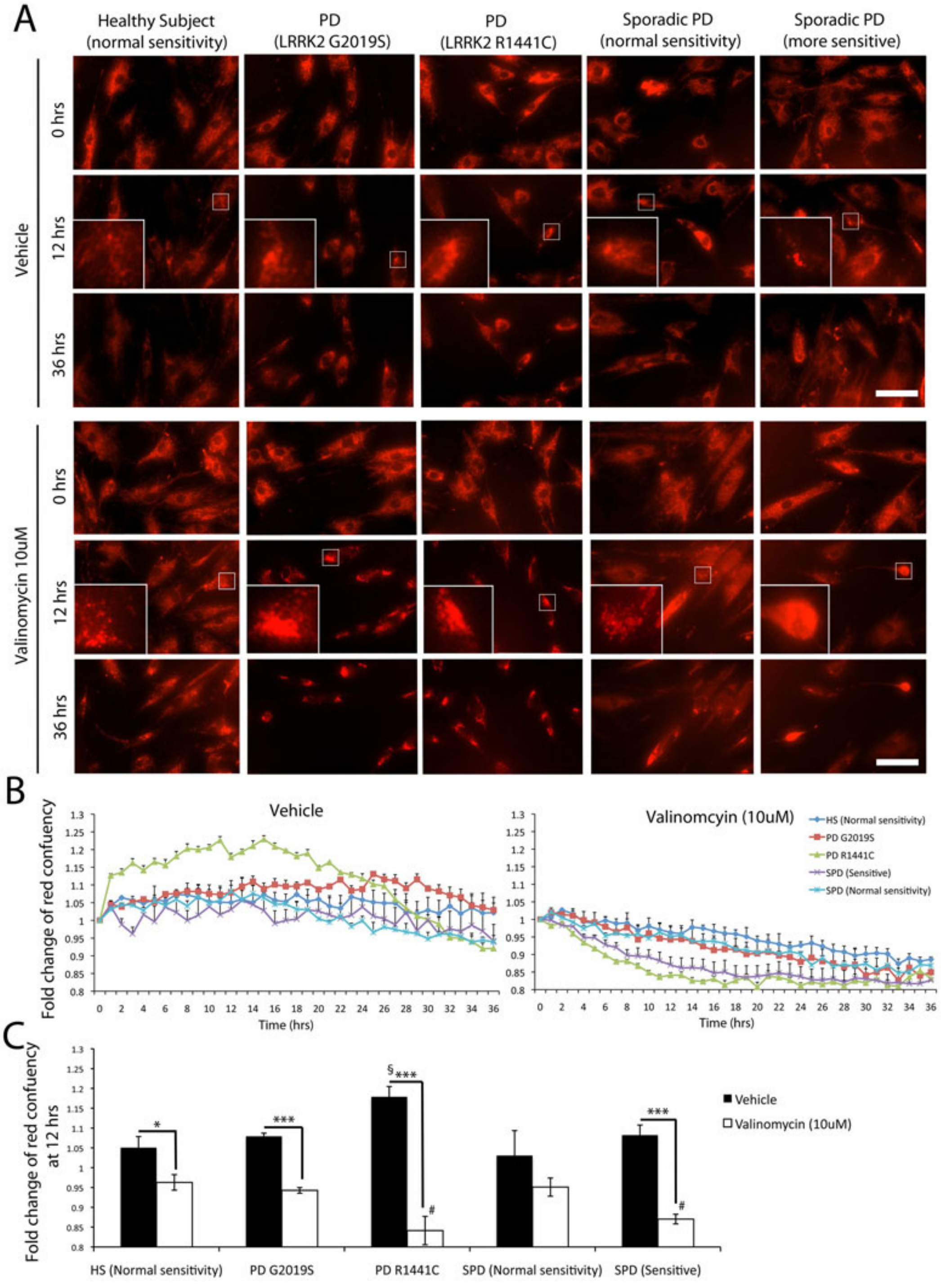

**Supplementary Fig. 2. Mitochondrial collapse around the perinuclear compartment in response to valinomycin occurs at a faster rate in more vulnerable sporadic PD patient fibroblast lines.** Mitotracker red was used to monitor the morphology of the mitochondrial network over 36 hrs, using live cell imaging (A). Fibroblast lines derived from healthy subject controls, mutant LRRK2 (G2019S) and sporadic PD patients showed a constant red object area size, over 36 hrs, under vehicle conditions (A-B). Fibroblast lines derived from mutant LRRK2 (R1441C) PD patients showed an initial increase in the size of the red object area under vehicle conditions (B). Conversely, all cell lines displayed a decrease in red object area, following the application of valinomycin (10μM) at 36 hrs post-treatment (B). Mitochondrial collapse could be observed in healthy subject control and mutant LRRK2 (G2019S) fibroblast lines, 12 hrs following the application of valinomycin, but was exacerbated in mutant LRRK2 (R1441C) and sensitive sporadic PD patient derived fibroblast lines at this time point (C). Graphs are annotated as $^{*}p<0.05$ and $^{***}p<0.001$ compared to vehicle conditions, $^{\$}p<0.05$ compared to healthy subject control (vehicle) and $^{\#}p<0.05$ compared to healthy subject control (valinomycin). Data was analyzed by a 2-way ANOVA with Dunnett's *post hoc* tests. Graphs are expressed at mean ± SEM. Scale bar = 100μM. N=4-5/ group.

**Supplementary Fig. 3**

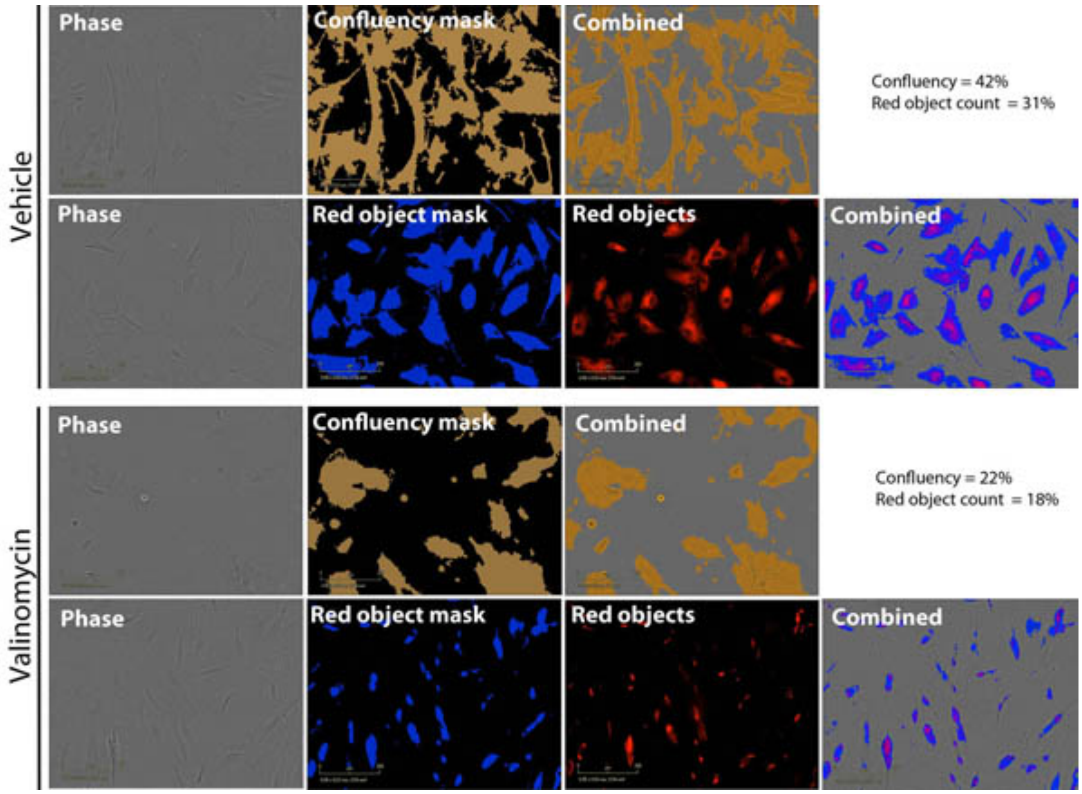


**Supplementary Fig. 3. Masks defining live cell-imaging analysis parameters.** User defined masks were optimized to determine the percent confluency based on automated phase contrast image analysis and the percent of Mitotracker red object area based on automated fluorescent image analysis. The efficiency of these parameters to determine confluency changes and mitochondrial collapse was tested by the application of valinomycin (40µM) for 12 hrs.

**Supplementary Fig. 4**

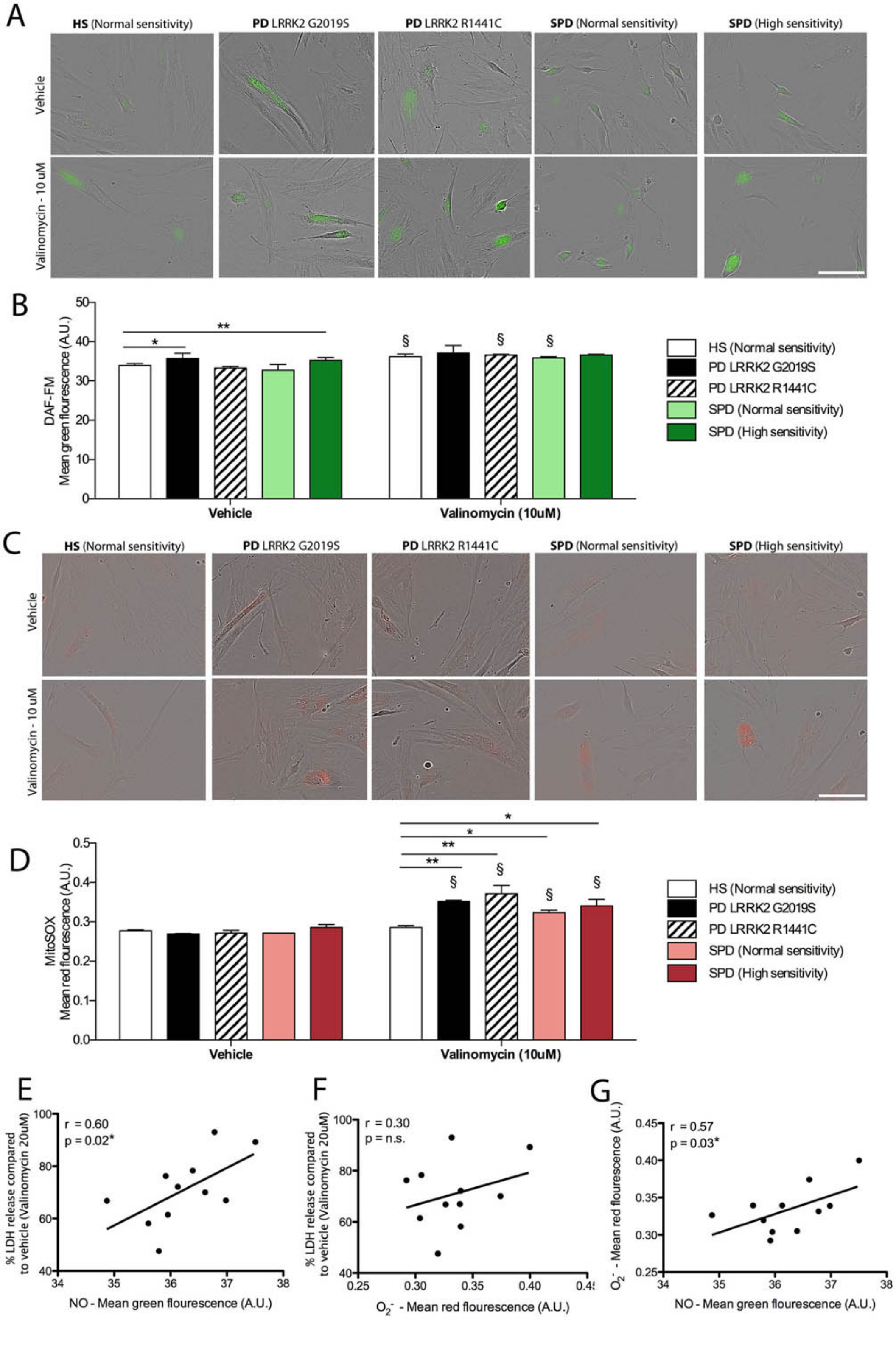

**Supplementary Fig. 4. Valinomycin causes the induction of nitric oxide and superoxide in mutant LRRK2 and sporadic PD patient derived fibroblast lines.** An increase nitric oxide species was observed in mutant LRRK2 (G2019S) and highly sensitive sporadic PD patient derived fibroblast lines under vehicle conditions (A-B). Nitric oxide levels were not exacerbated further by valinomycin treatment (10µM), given for 24 hrs, in mutant LRRK2 (G2019S) and highly sensitive sporadic PD patient derived fibroblast lines with valinomcyin application, yet were increased in all other lines (A-B). In contrast, superoxide levels did not differ between groups under vehicle conditions and were increased in all PD lines following valinomycin exposure (C-D). The increase in nitric oxide species in sporadic PD patient derived fibroblast lines at 10µM of valinomycin was positively correlated with valinomcyin-induced toxicity observed at 50µM (E). No correlation was observed between valinomycin mediated cell death and superoxide levels (F). Superoxide levels and nitric oxide levels in sporadic PD patient derived fibroblast lines were positively correlated (G). Graphs are annotated as $^{*}p<0.05$ and $^{**}p<0.01$ compared to healthy subject control lines and $^{\S}p<0.05$ compared to vehicle conditions. Data was analyzed by 2-way ANOVAs with Dunnett's *post hoc* tests or Pearson's correlation. Graphs are expressed at mean ± SEM. N=4-5/ group. Scale bar = 200µM.

## Supplementary Fig. 5

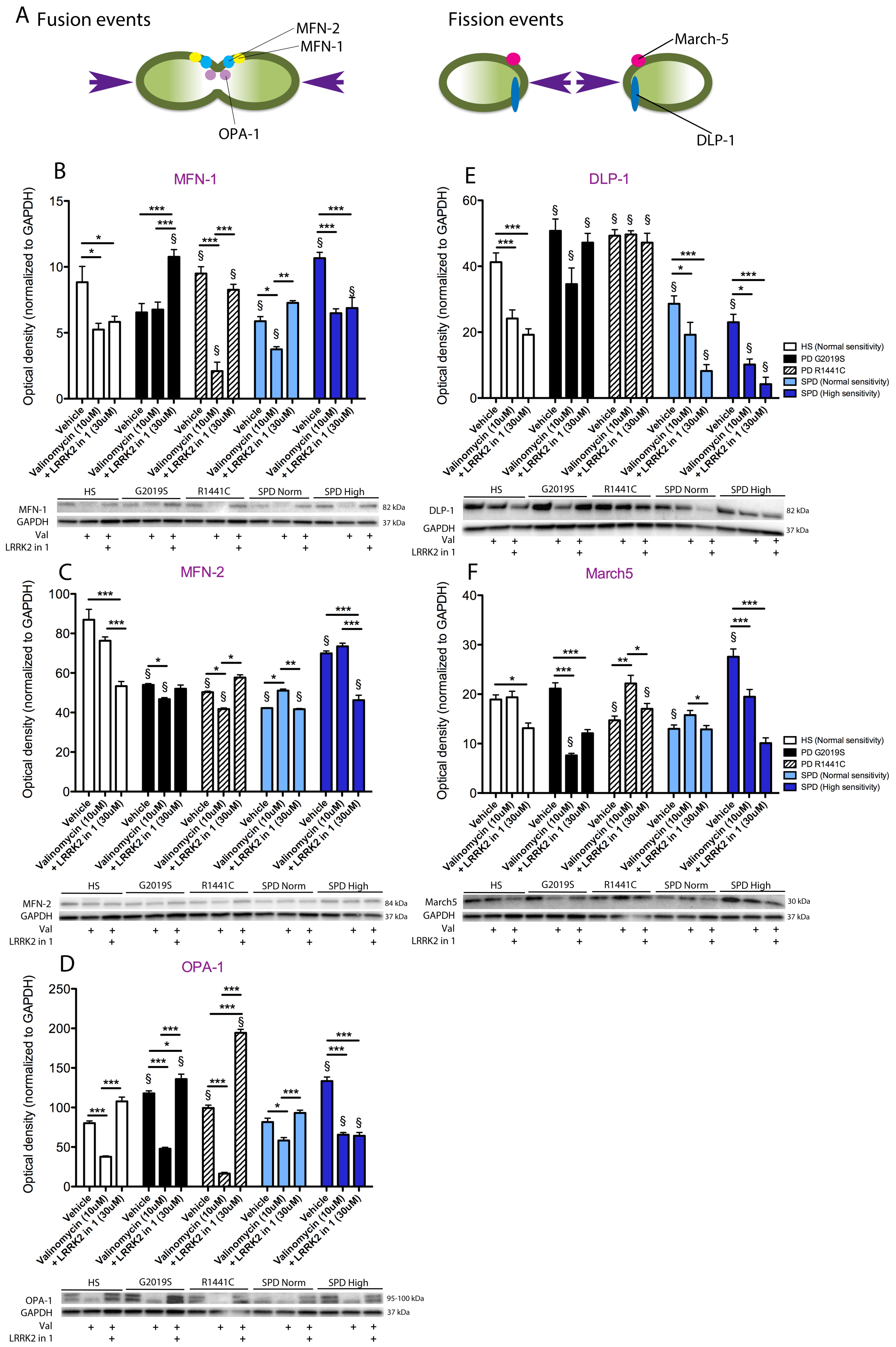

**Supplementary Fig. 5. LRRK2-in-1 differentially regulates key mitochondrial fission and fusion proteins between mutant LRRK2 and sporadic PD patient fibroblast lines.** Mitochondrial fission and fusion events are mediated by defined mitochondrial-associated proteins (A). Protein levels of the mitochondrial fusion protein Mfn1 were increased in mutant LRRK2 (R1441C) and sensitive sporadic PD patient derived fibroblast lines, compared to healthy subject control lines under vehicle conditions, yet were decreased in less sensitive sporadic PD patient derived fibroblast lines (B). 24 hrs of valinomycin exposure caused a reduction in Mfn1 protein levels in mutant LRRK2 (R1441C), sporadic PD and healthy subject control derived fibroblast lines (B). Conversely, the co-application of valinomycin and LRRK2-in-1, for 24 hrs, increased protein levels of Mfn1 in mutant LRRK2 (G2019S & R1441C) and less sensitive sporadic PD patient derived fibroblast lines, compared to healthy subject control lines (B). Protein levels of the mitochondrial fusion protein Mfn2 were reduced in mutant LRRK2 (G2019S & R1441C) and less sensitive sporadic PD patient derived fibroblast lines, compared to healthy subject control lines under vehicle conditions (C). Valinomycin caused a further reduction in Mfn2 levels in mutant LRRK2 (G2019S & R1441C) PD patient derived fibroblast lines, which could be restored by LRRK2-in-1 application (C). In contrast, LRRK2-in-1 reduced levels of Mfn2 in healthy subject control and sporadic PD patient derived fibroblast lines (C). At baseline there were elevated levels of the mitochondrial fusion protein Opa1 in mutant LRRK2 (G2019S & R1441C) and highly sensitive sporadic PD patient derived fibroblast lines, compared to healthy subject control lines (D). Valinomycin reduced the protein levels of Opa1 in all cell lines (D). The co-application of valinomycin and LRRK2-in-1 caused a restoration of Opa1 levels, however this was not observed in highly sensitive sporadic PD patient derived fibroblast lines (D). The mitochondrial fission protein Dlp1 was increased in LRRK2 mutation carrying lines yet decreased in sporadic PD lines under vehicle conditions (E). Valinomycin treatment reduced protein levels of Dlp1 in all fibroblast lines (E). The valinomycin-induced decrease in Dlp1 was not rescued by LRRK2-in-1 (E). At baseline there were elevated levels of the mitochondrial fission protein MARCH5 in highly sensitive sporadic PD patient derived fibroblast lines, compared to healthy subject control lines (G). MARCH5 levels were decreased following valinomycin application in mutant LRRK2 (G2019S) and highly sensitive sporadic PD patient derived fibroblast lines and were increased in mutant LRRK2 (R1441C) and less sensitive sporadic PD patient derived fibroblast lines (G). LRRK2-in-1 application was able to restore valinomycin-induced increases in MARCH5 protein levels (G). Graphs are annotated as $^{\S}p<0.05$ compared to healthy subject controls and $^{*}p<0.05$, $^{**}p<0.01$ and $^{***}p<0.001$ compared to vehicle conditions, as analyzed by a multivariate ANOVAs with Dunnett's *post hoc* tests. Graphs are expressed at mean ± SEM. N=3/ group.

**Supplementary Fig. 6**

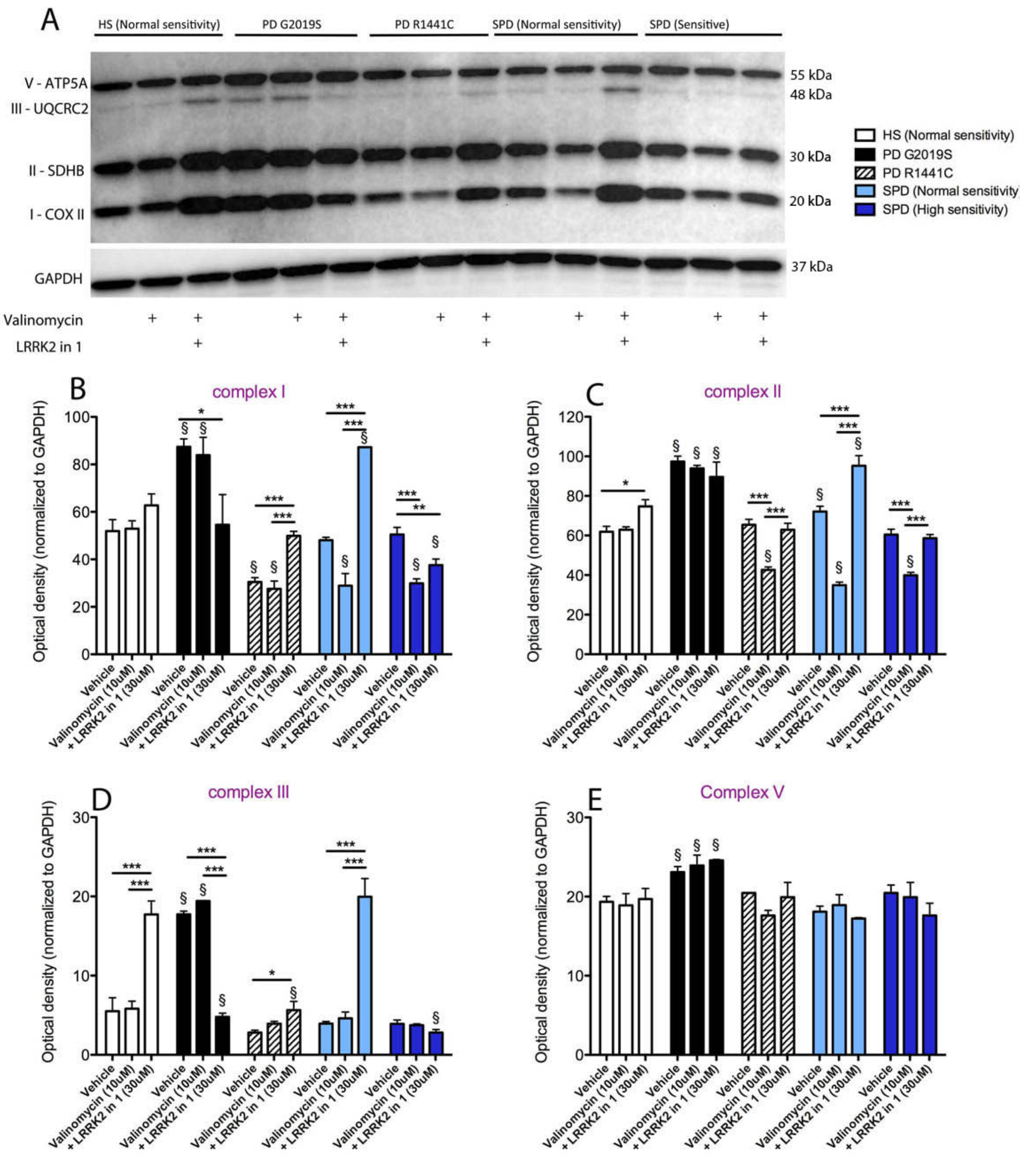

**Supplementary Fig. 6. LRRK2-in-1 differentially regulates mitochondrial complex levels following valinomycin exposure.** At baseline protein levels of mitochondrial complex I were highest in mutant LRRK2 (G2019S) PD patient derived fibroblast lines, compared to healthy subject control lines, yet reduced levels were observed in mutant LRRK2 (R1441C) lines (A-B). Following valinomycin (10µM) exposure for 24 hrs levels of complex I were reduced in highly sensitive sporadic PD patient derived fibroblast lines (A-B). The co-application of valinomycin and LRRK2-in-1 for the same duration caused an increase in complex I protein levels in LRRK2 mutation carrying (R1441C) and sporadic PD fibroblast lines, yet caused a reduction in mutant LRRK2 (G2019S) lines (A-B). Complex II protein levels were significantly increased in mutant LRRK2 PD patient derived fibroblast lines, under vehicle conditions, compared to healthy subject control derived lines (A, C). The application of valinomycin significantly reduced complex II levels in LRRK2 mutation carrying (R1441C) and sporadic PD patient derived fibroblast lines, which was restored by LRRK2-in-1 treatment (A, C). At baseline protein levels of mitochondrial complex III were highest in mutant LRRK2 (G2019S) PD patient derived fibroblast lines, compared to healthy subject control lines and levels did not change following valinomycin exposure (A, D). LRRK2-in-1 treatment significantly reduced levels of complex III in mutant LRRK2 (G2019S) PD patient derived fibroblast lines, yet increased levels in mutant LRRK2 (R1441C), less sensitive sporadic PD and healthy subject control derived fibroblast lines (A, D). Complex V protein levels were significantly higher in mutant LRRK2 (G2019S) PD patient derived fibroblast lines and did not change with either valinomycin exposure or LRRK2-in-1 treatment (A, E). Graphs are annotated as $^{\S}p<0.01$ compared to healthy subject controls and $^{*}p<0.05$, $^{**}p<0.01$ and $^{***}p<0.001$ compared to vehicle conditions, as analyzed by a multivariate ANOVAs with Dunnett's *post hoc* tests. Graphs are expressed at mean ± SEM. N=3/ group

**Supplementary Fig. 7**

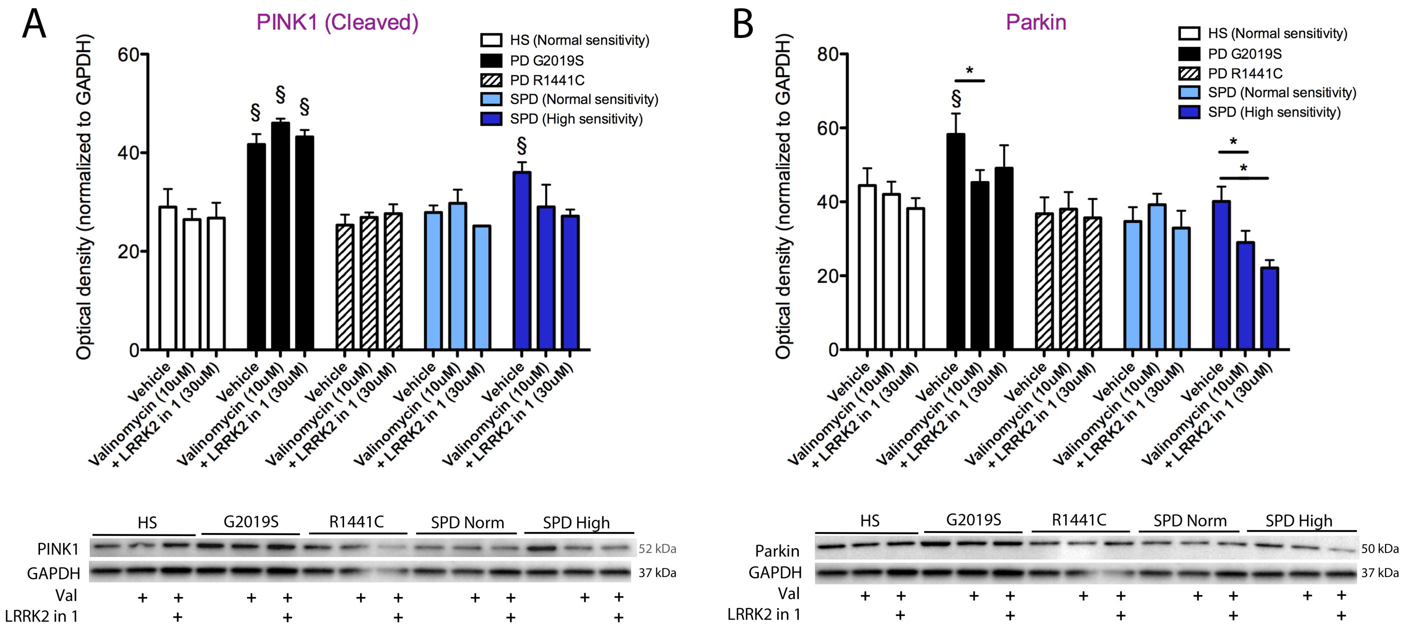


**Supplementary Fig. 7. Cleaved PINK1 and Parkin protein levels in PD patient derived fibroblasts.** Cleaved PINK1 protein levels were significantly increased in mutant LRRK2 (G2019S) PD patient derived fibroblast lines irrespective of the treatment group (A). Sensitive sporadic PD patient derived friboblast lines also displayed increased levels of cleaved PINK1 under vehicle conditions (A). The protein levels of Parkin were significantly elevated at baseline in mutant LRRK2 (G2019S) lines, compared to healthy subject controls at baseline (B). Protein levels of Parkin were significantly reduced in mutant LRRK2 (G2019S) and sensitive sporadic PD fibroblast lines following the application of valinomycin at 10uM for 24 hrs (B). Valinomcyin and LRRK2-in-1 co-treatment had little effect on Parkin levels in mutant LRRK2 (G2019S) and sensitive sporadic PD groups (B). Graphs are annotated as $^{\S}p<0.05$ compared to healthy subject controls and $^{*}p<0.05$ compared to vehicle conditions, as analyzed by a multivariate ANOVAs with Dunnett's *post hoc* tests. Graphs are expressed at mean ± SEM. N=3/ group.

**Supplementary Fig. 8.**

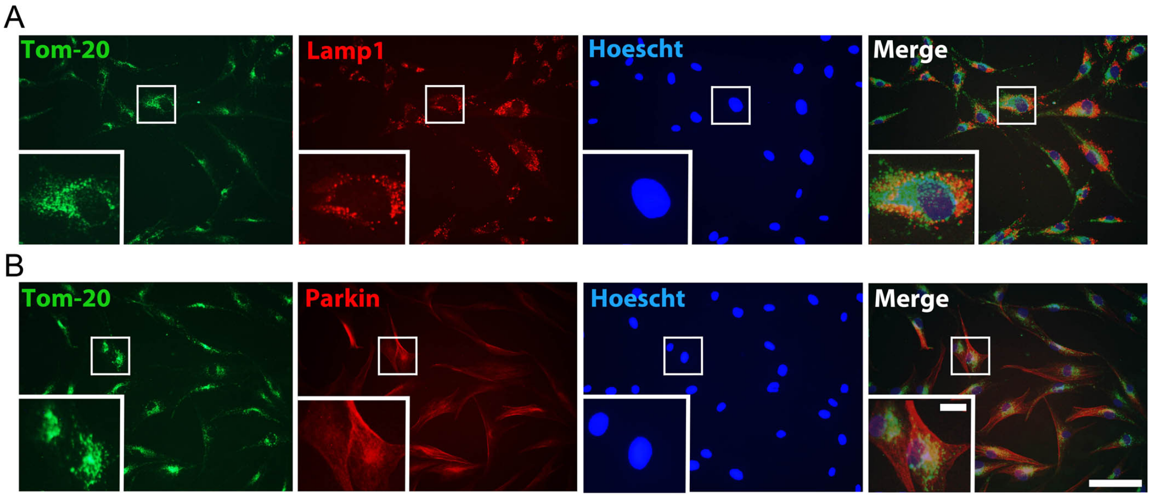


**Supplementary Fig. 8. The effect of LRRK2-in-1 on lysosome morphology and Parkin distribution.** The application of LRRK2-in-1 (30µM) alone, given for 24 hrs, caused increased formation of LAMP1 positive lysosomes throughout the cytoplasm (red), yet does not change the morphology of Tom20 positive mitochondria (green), (A). There was no apparent increase in mitochondria-lysosome colocalization by the application of LRRK2-in-1 alone (A). LRRK2-in-1 treatment alone did not change the cytoplasmic localization of Parkin and did not cause the formation of Parkin rings (B). Low magnification scale bar = 200µM and high magnification scale bar = 50µM. N=3/ group

**Supplementary Fig. 9.**

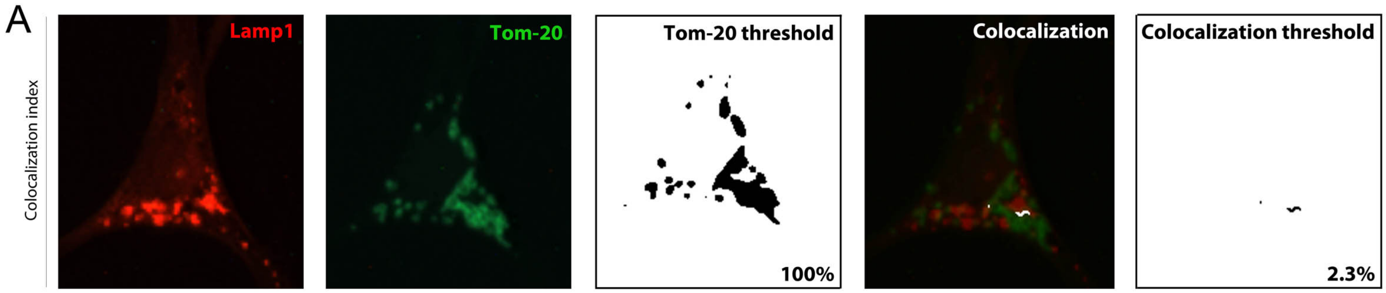


**Supplementary Fig. 9. Masks used to measure mitochondria and lysosome colocalization in fibroblast lines.** The percent area of overlap between LAMP1 positive lysosomes (red) and Tom20 positive mitochondria (green) within a fibroblast can be thresholded and subtracted from the total thresholded area of Tom20 coverage (A).
